# A Week in the Life of the Human Brain: Stable States Punctuated by Chaotic-Like Transitions

**DOI:** 10.21203/rs.3.rs-2752903/v1

**Published:** 2023-03-30

**Authors:** Maxwell B. Wang, Max G’Sell, R. Mark Richardson, Avniel Singh Ghuman

**Affiliations:** 1. Neuroscience Institute, Carnegie Mellon University, Pittsburgh, PA 15213, USA; 2. Machine Learning Department, Carnegie Mellon University, Pittsburgh, PA 15213, USA; 3. Department of Neurological Surgery, University of Pittsburgh, Pittsburgh, PA 15213, USA; 4. Medical Scientist Training Program, University of Pittsburgh and Carnegie Mellon University, Pittsburgh, PA 15213, USA; 5. Department of Statistics and Data Science, Carnegie Mellon University, Pittsburgh, PA 15213, USA; 6. Department of Neurosurgery, Massachusetts General Hospital, Boston, MA 02114, USA; 7. Harvard Medical School, Boston, MA 02115, USA; 8. Center for the Neural Basis of Cognition, University of Pittsburgh and Carnegie Mellon University, Pittsburgh, PA 15213, USA; 9. Center for Neuroscience at the University of Pittsburgh, Pittsburgh, PA 15213, USA

## Abstract

Critical neurocognitive processes, such as performing natural activities and fluctuations of arousal, take place over minutes-to-days in real-world environments. Here we harness the opportunity to study brain dynamics during real-world behavior continuously for 3–12 days using multi-electrode intracranial recordings in twenty humans. During this time, participants engaged in natural activities, including interacting with friends, family, and staff, watching TV, sleeping, etc., with simultaneous neural and video recordings. We found that brain network dynamics predicted neurocognitive phenomena such as circadian rhythm, arousal, and multiple aspects of behavior (socializing, watching a screen, etc.). The individual functional networks, as well as their pairwise interactions, possessed simple and stable dynamic properties that were conserved over days. In contrast to single or paired network behavior, the mixture of all functional networks showed patterns of “punctuated equilibrium”: periods where networks would remain in stable states that corresponded to behavior and were interrupted by transitory bursts that were difficult to predict, displayed chaotic characteristics, and coincided with behavioral transitions. Brain state statistics displayed power laws characteristic of critical dynamics that are a trait of systems where complexity emerges from simple and stable building blocks. These results indicate that the complex and flexible brain dynamics that underpin real-world behavior are an emergent property of mixtures of individual, stable networks with simple dynamics.

Many important neurocognitive processes take place on the order of minutes to days in dynamic, ever-changing “real” environments. Behaviorally, we transition between tasks like reading and talking to friends over minutes to hours. Neurobiologically, the interaction between someone’s brain and body is driven by hormones, sympathetic, and parasympathetic drivers related to processes like arousal and circadian rhythms that fluctuate over a similar timescale^1^. However, the vast majority of what we know about human brain activity is based on studies that record neural signals on the scale of milliseconds-to-seconds while participants perform well-controlled tasks or rest in an artificial neuroimaging environment.

Some studies have analyzed brain state dynamics over minutes in a single sitting or by repeatedly sampling a few minutes per day spread out over days to months in an artificial setting using functional neuroimaging^2–10^. Longitudinal snapshots of a few minutes over days have also been studied in real-world settings, though typically associated with structured tasks or treatment interventions^11,12^. While much has been learned about human brain network dynamics in these types of studies, we still do not know how the brain continuously evolves over hours-to-days in real-world settings during natural behavior.

To assess human brain network dynamics in a real-world setting continuously over days, we leveraged chronic intracranial recordings in neurosurgical participants (80–126 electrodes implanted per participant) undergoing treatment for epilepsy (Figure 1 and Supplemental Figure S1). We examined brain network dynamics from neural recordings in twenty humans for between 75 to 283 hours (near-continuous recordings across approximately 3–12 days). During this time, the participants were confined to the hospital but would freely socialize with friends, family, and staff, interact with digital devices, sleep, watch TV, and perform other volitional natural behaviors while under neural and video monitoring. Using these data, we built all-to-all electrode functional connectivity matrices (partial connectomes) every five seconds across the entire recording session (Figure 1), divided these connectivity matrices into data-driven networks, and removed electrodes and activity related to each participant’s seizure onset zone and propagation. We then asked, what are the properties of brain network dynamics during continuous, natural behavior over the week?

Notably, the brain must balance competing demands of flexibility that allows people to react to changing cognitive demands while maintaining anatomical stability of networks and systems. Thus, we particularly asked what features of brain network dynamics changed rapidly, what aspects were consistent from hour-to-hour and day-to-day, and how these dynamics related to natural behavior. We investigated these questions over increasing spatial scale: starting with individual areas (functionally defined “parcels”), to the dynamics of covarying sets of parcel activity (“network components” acknowledging that we only have partial brain coverage in individual participants), to patterns of pairwise interactions between different network components, and finally to how the brain forms states and transitions during its week-long trajectory through a space defined by all network components (mixtures of all networks).

## Functional parcels show temporal consistency over days and reveal anatomic trends

Before studying how the whole measured brain evolves over the course of a week, we started by breaking it down into smaller pieces and studying how those pieces change over the week in isolation. We used a data-driven approach to identify small groups of tightly connected electrodes that made up coherent functional brain parcels (we use the term brain “parcels” rather than brain “areas” because brain areas are traditionally defined based on anatomical landmarks, and these brain parcels are anatomically compact but defined based on similarity/high coherence of the neural activity within a parcel). Our first question was whether these parcels showed stable temporal characteristics over the week: e.g., if a brain parcel fluctuates quickly on one day relative to other parcels, does it do so on other days as well?

After removing an hour before and after ictal (seizure) events as determined by the clinical team, the coherence between all pairs of electrodes in a participant was calculated every five seconds over five frequency bands: theta (θ: 4–8Hz), alpha (α: 8–12Hz), low beta (β_l_: 14–20Hz), high beta (β_u_: 20–30Hz), and gamma (γ: 30–70Hz). The electrodes were parcellated into tightly connected groups of electrodes (a coherent functional brain “parcel”). The parcel assignments remained very stable throughout the week (Supplementary Figure S2), which allowed us to define one set of parcels for the entire week. Parcels consistently contained electrodes that were anatomically close together, hence our description of this process as “parcellation” of the brain. To remove seizure-related activity from our analyses, we first removed any parcels associated with participants’ seizure onset zone and early propagation. We further removed any activity from the remaining non-seizure-related parcels that correlated with activity from parcels within the participants’ seizure onset zones outside of ictal periods to ensure that any potential pathological activity (such as responses related to interictal activity) was removed from our analysis. We found that the coherence within each parcel showed specific dynamical patterns or trajectories that would be repeated over different hours and days of data (Figure 2A and Supplementary Figure S3). This dynamical stability can be quantified by how slowly their autocorrelation curves decayed (timescales). We found that timescale differences between parcels were preserved over time, indicating that parcels with relatively faster or slower timescales would remain so throughout the week. Specifically, the autocorrelation magnitude at one hour and the timescale of how quickly autocorrelation decayed showed reliable differences between parcels that were conserved over different six-hour time blocks throughout the week (Supplementary Figure S3).

These parcel-to-parcel differences were linked to anatomical trends over all twenty participants by assigning each parcel to one of five lobes (frontal, temporal, parietal, occipital, and basal ganglia) and one of six canonical fMRI networks (“default mode”, “dorsal attention”, “salience”, “somatomotor”, “control”, and “visual” as defined in^13^) dependent on which lobe/network it had the most overlap with (parcels with no clear overlap were not considered for this analysis). Parcels in the default mode network and basal ganglia consistently showed higher autocorrelation magnitude and longer autocorrelation decay timescales across our participants while parcels of the salience network showed shorter timescales (Figure 2B). These findings demonstrate an intrinsic stability in neural dynamics that separates “slow” from “fast” brain parcels over minutes to hours. A temporal hierarchy, typically measured using autocorrelation, has been hypothesized in the brain with transmodal (e.g. default mode network) systems being the slowest, integrating over multiple seconds^2,3,14–17^. Our findings extend the observation of slow default mode network dynamics to minutes and hours during natural behavior in a real-world setting.

## Network components form dynamical relationships and joint distributions that are preserved over days

After investigating how parcels of the brain would act on an individual basis, we wanted to understand how they interacted with each other. As many parcels were highly co-linear with each other (Supplementary Figure S17), we decided to group co-linear parcels into networks*. After finding that those networks also possessed consistent timescales as individual parcels did (Supplementary Figure S5), we examined how these networks interacted with one another.

More specifically, we used robust principal components analysis to identify parcels and frequencies that covaried with each other, defining each principal component as a “network component” that captured the overall connectome dynamics in a data-driven fashion while reducing the dimensionality of the dataset^18,19^ (we use the term “network components” because the recordings did not have full brain coverage and therefore these covarying parcels are components of brain networks). The activation of each network component during a window was defined as the weighted average of the parcel coherences within a network component (dot product between network activation and principal component weights). These network component activations showed consistent temporal behavior over time as individual functional regions did (Supplemental Figure S5).

After investigating individual network components, going up one step in spatial scale, we asked whether the activity of pairs of network components could be reliably linked to one another. For each day, we calculated the joint distribution between all possible pairs of network component activations and asked whether this joint distribution was both reliably preserved across the week and indicated significant non-independent and/or non-linear relationships (while principal components will group features with linear relationships together, it will not do the same for non-linearities). More specifically, we calculated the distance between the joint distributions on different days of recordings versus the distance between these distributions and an independent null (more detail in Methods). The joint distribution between brain networks covered characteristic areas in the space that were well preserved over days, indicating that brain networks “dance” with one another in idiosyncratic ways. Some had antagonistic relationships where one network appears to suppress another; some would behave as if one network “gated” the other – inactivity in one network (in other words low coherence within contained parcels) would mandate inactivity in another. For example, the “V” shaped patterns in Figure 3A indicate that either both networks would be inactive together, or when one was active, the other would either be positively active or negatively active (but not inactive). All participants possessed several networks that showed such pairwise interactions (Figure 3 and Supplemental Figure S6), indicating that not only do individual network components have consistent dynamics, but they also have consistent pair-wise interactions.

## Network components predict both physiology and behavior

One critical question is whether these network dynamics are related to neurophysiology and behavior. We specifically looked at how linear combinations of network components correlated to circadian rhythm (time of day), predicted arousal (heart rate), and classified behavior (video recordings).

We took the first half of the week for each participant and used canonical correlation analysis (CCA)^20^ to identify a network mixture that maximized correlation to time of day (CCA was used over regression to allow encoding time of day via phase). We tested this mixture in the second half of the week using permutation testing (out of sample validated correlation) and found that 11 of 20 participants had a network mixture significantly linked with circadian rhythm (Figure 4A and Supplementary Figure S7). Notably, six of the nine participants that were not linked to circadian rhythm had notes in their clinical file indicating various sleep disturbances such as nighttime-awakening seizures, intentional sleep deprivation for clinical purposes, or difficulty sleeping; suggesting these participants had disrupted circadian rhythms due to sleep issues during the week.

Seven participants had sufficiently clean electrocardiogram (EKG) signals that were used to track heart rate. Heart rate is strongly correlated with the degree of arousal and is used here as an approximator^21^. We used L1-regularized^22^ regression over the first half of the week to identify a mixture of networks that predicted heart rate and tested it on the remaining half (Figures 4B and Supplementary S8). We found that six of the seven participants had network components that were significantly associated with arousal.

Nine participants had video recording monitoring available throughout the week that was of sufficient quality to determine what the participant was doing throughout each day. We randomly selected two days from each participant and labeled times when the participant was watching a digital screen, socializing with another person, or physically manipulating a held object. These three behavioral labels were not mutually exclusive. We trained L1-regularized logistic classifiers on one day to identify a mixture of network components associated with each behavior and then tested them on the second day. All participants possessed network components that were significantly associated with behavior; two participants are shown in Figure 4C and all participants are shown in Supplementary S9.

Taken together, these three tests show that network components predict both physiological metrics and behavior that was replicated on multiple days.

## Mixtures of network components form a punctuated equilibrium of stable states that coincide with behavior

After we found that mixtures of network components were linked to behavior and physiology, we wanted to understand how the status of all network components changed throughout the week. Up to this point, we have investigated how individual, or pairs of network components change over time. This is analogous to studying how a hummingbird transitions between hovering and flitting between flowers by only observing their movement in one or two spatial dimensions at a time. Just as how a complete picture of a hummingbird’s flight patterns requires a full three-dimensional space, we studied brain trajectories through a high-dimensional neural space, with the dimensions defined by each of the network component’s activity.

Figure 5A (left) shows the velocity of the brain over one participant’s week: how quickly the participant’s brain network activations changed every five seconds. This is analogous to the speed of a hummingbird’s movement: high when it’s flying, low when it’s hovering. In this case, hovering means the network activations of someone’s brain are remaining relatively steady while high velocity flight indicates that the network activations are changing rapidly. Quantitatively, velocity was calculated by taking the vector of all network activations from one time window and calculating the Euclidean distance between it and the corresponding vector from the next time window^†^. The results indicated that periods of stability (states), times of low velocity lasting for minutes-to-hours, were interspersed with bursts of dynamic behavior marked by high velocity for periods lasting up to minutes (state transitions).

To quantify that those times of high velocity occurred in “bursts,” we tested the time between windows that fell into the top 1% or 10% of speed in each participant (Supplementary Figure S18). We found that windows with high velocity tended to occur temporally adjacent or close to one another (bursts of high velocity) at significantly higher rates than if they occurred randomly via homogeneous Poisson process, just as a hummingbird’s high velocity periods occur in bursts. The rest of the analyses in this study examined the nature and statistics of these state transitions, defined as temporally contiguous bursts of high velocity over which the brain is continuously reconfiguring itself^23^. Long periods of stable states interspersed with bursts of high speed transitions is characteristic of “punctuated equilibrium”^24^, an observation that many systems and processes in nature, particularly ones that involve adapting to a dynamic environment^24^, do not undergo steady gradual change but rather periods of stability interrupted by rapid bursts of change.

The correspondence between behavioral and brain network transitions was next used to assess the relevance of these brain network state transitions. Specifically, we calculated the median time between a participant’s behavioral changepoint (any point one of their three labeled behaviors changed) and the nearest identified neural state transition. We compared this to the expected time if there were no relation between the two using permutation testing. We found that for every participant, the median time was smaller than the expected time using a paired t-test (Figure 5C, p<1e-4). This result demonstrates that brain network transitions and behavioral transitions coincide with one another in time.

## Brain network transitions are circuitous, unpredictable, and chaotic

After we found that stable networks and pairwise interactions could lead to the formation of neural states that lasted for minutes to hours and were associated with behavior, we asked how the brain transitions from one state to another. Specifically, does the brain take relatively straight trajectories when transitioning between states, as a hummingbird does when it moves between one hovering location to another, or are brain trajectories more circuitous. We visualized the transitory bursts of high velocity and stable states in Figure 5A (left) onto a t-distributed stochastic neighbor embedding (TSNE) representation of the week-long data in Figure 5A (right). TSNE is a data visualization technique that shows a two-dimensional representation of the data that preserves the distance between points plotted; thus, points that are close together are ones that have similar brain network activation vectors^25^. Visually, these transitions appeared to take very indirect trajectories. Instead of the brain transitioning directly from one stable state to another, it would appear to “wander around” and explore several possible intermediate states of various network activations or deactivations before stabilizing into the destination.

We quantitatively tested this by comparing the total “distance” traveled by the brain during a transition trajectory (the sum of the distance traversed during each step of the trajectory during a transition) compared to the net “displacement” (the distance between the starting and end states). For a straight-line trajectory (a hummingbird flying directly from one flower to another), the distance equals the displacement. Transition trajectories were defined as periods of high velocity surrounding detected change points (more details in Methods). All quantitative analyses were done in the original network activation space, TSNE was only used for visualization. Transitions across all participants showed total distances several times larger than the net displacement on average, indicating they were taking indirect routes between states (Figure 5C). Notably, the ratio of the distance to displacement was larger during transitions than during stable states, indicating that between-state trajectories were more circuitous than within state trajectories.

These results demonstrate that transition trajectories are indirect, but indirect routes can still be consistent each time (e.g., when the brain transitions from stable states A to B does the path taken remain the same each time?). To assess their consistency, we compared transitions with the same start and end point both to transitions with the same start point, but different end points, and to transitions with different start points, but the same end point (Figure 5D and Supplementary Figure S10). If transition trajectories were repeated over the course of the week, the distance between pairs of transitions that started and ended in similar states (repeated transitions) would remain small compared to the distance between pairs of transitions that started in the same state but ended up in differing ones. On the contrary, the results indicated that transitions with the same end state were no more similar than transitions with different end states until 40–50% completion (Figure 5D). When they separated, the Cohen’s d effect size of this divergence remained below 1 (less than one standard deviation apart) until 3/4 of the transition was complete. Thus, even if two trajectories had the same start and end point, the trajectories were typically very different from each other given that the distance between them was comparable to the distance between trajectories with different ending points for much of their journey.

Supporting the idea of diverse and hard to predict state transitions, autoregressive prediction error increased during transitions compared to within state periods (Figure 5D bottom). Autoregressive predictors were trained on half the week and tested on the remaining half, demonstrating lower error at predicting “within-state” relative to “between-state” movement. In addition, transitions exhibited increased chaoticity compared to within-state dynamics, measured using the 0–1 chaos test^26^. Taken together, these results indicate that when the brain transitions from one stable state to another, not only would it rapidly explore a large set of intermediate states before settling down into the destination, but that these intermediate states seemed to be chosen in a disorganized, chaotic-like manner.

## State transitions consistently occurred via power laws

We sought to reconcile the picture of simple neural networks with reliable pairwise interactions against the complex and chaotic-like transitory bursts that periodically spread throughout the brain. Many hypotheses investigating how complex neural processes can emerge from simple systems are distinguished by the distribution of various metrics summarizing salient features of their dynamics. Up to this point, we have primarily investigated the average value or variance of metrics on the brain’s long-term dynamics such as timescales, mean chaoticity, or the predictability of neurocognitively interesting variables. Here we investigated the distribution of two metrics of neural state transitions: how large they are and how frequently they occur.

Figure 5E shows the distribution of the net displacement of these transitions and the time between transitions. These distributions visually followed power laws (linear on log-log plots), indicating that while transitions at first glance appeared to be disorganized and chaotic-like, there were overlying patterns governing the transition statistics that remained consistent from participant to participant. This overall dichotomy is illustrated in Figure 6.

We quantitatively tested this finding using a combination of likelihood ratio and Kolmogorov-Smirnov tests. We tested the likelihood that each participant’s distribution came from a power law distribution vs exponential or log-normal distributions. The results indicate that power law distributions were the most likely fit in the transition size distribution of 16 of 20 participants (p=0.03) and the most likely fit in all 20 time between transition distributions (p=4.8e-5). We used Kolmogorov-Smirnov tests to see if we could reject a null hypothesis that each participant’s distribution came from a power law distribution. These tests failed to reject in 17/20 participants’ transition size distribution and in 20/20 participants’ time between transition distributions. Together, these tests support that the brain network state transitions follow power law distributions (Figure 5F) that occur when someone’s behavior also changes (Figure 5B), linking power laws of the brain to real-world behavior and cognition. These results are consistent with the “critical brain hypothesis” which claims complex group-level behavior can emerge from simple and stable local activity in systems perched between order and disorder^27^.

## Discussion

The results of this study demonstrate key properties of how the brain continuously evolves over long timescales by recording roughly a week of near-continuous intracranial recordings in each of twenty human participants. The results indicate a dichotomy between relatively “local” brain dynamics that remained remarkably stable over time whereas overall brain trajectories and state transitions were varied and chaotic.

Individual parcels and networks had characteristic time constants and trajectories that were well preserved across multiple days (Figure 2). Pairs of parcels and networks displayed characteristic, non-random “dances” that also remained stable over time (Figure 3). In contrast, the global mixture of brain networks displayed stable states punctuated by chaotic transitions between them (Figure 5). When the brain entered a stable state, the balance of its networks would remain relatively consistent for periods lasting from minutes to hours. We found that by using these networks, we could predict somebody’s behavior (such as were they interacting with a screen or talking to a friend) and their physiological status (circadian rhythm and arousal; Figure 4). When somebody’s behavior changes, potentially reflecting environmental or cognitive changes such as deciding to switch from reading a book to watching television or a friend walking into their room and beginning a conversation, their brain state would also change^28^. These transitions did not occur by the brain traveling from one state directly to the next but rather by circuitous, difficult to predict, and chaotic-like routes where the brain would explore many intermediate stages before settling down into a new stable state (Figure 5). While these transitions were difficult to predict and remarkably varied, their overall statistics (size and frequency) consistently followed power law distributions across participants (see Figure 6 for a summary).

Stable brain states interrupted by chaotic-like transitions are akin to punctuated equilibrium in evolutionary biology. Evolutionary history does not only show steady and gradual development but also alternates between periods of stability and transient bursts of rapid change^29^. These transitory periods are relatively disorganized: in evolution, these bursts involve phylogenetic “explosions” that generate multiple species or variants that quickly undergo environmental selection^24^. This also generalizes to large human organizations and political systems^30^. In most successful businesses, innovation efforts typically come in waves where an organization will explore several possible opportunities before settling on a much smaller number to develop^31^. Indeed, some studies suggest that punctuated equilibrium is a hallmark of relatively efficient group decision-making^32^. In our participants, their long-term neural dynamics would typically explore a large space before settling into stable behaviorally associated states.

Punctuated equilibrium may be how our brains deal with the chaotic and unpredictable real-world environment. Just as Darwinian evolution does not know the true end-optimal genetic state for the current ecosystem, our brains do not intrinsically know what will happen an hour in the future. Generating unpredictable and chaotic exploratory trajectories may be a key strategy of how our brains react to changing environments.

How can the brain quickly generate these chaotic-like trajectories from simple and stable networks that are conserved over the week? While these trajectories were remarkably varied and difficult to predict, their overall statistics consistently followed power laws across our participants, indicating some conserved pattern.

One possible explanation for this pattern is self-organized criticality (SOC) from statistical mechanics. SOC argues that systems made up of many small actors with simple interactions can manifest complex group-level behavior by oscillating around a “critical point” – transition points between phases of matter or system behavior^33^. For example, while freezing and melting will spread simply from molecule to molecule to lead to ice cubes or simple liquids, being held in a state fluctuating between melting and freezing dynamics records the cumulative history of all these oscillations which leads to the complex and unique shapes of snowflakes. In the case of the brain, theories of SOC posit that brain regions and networks are held in a state balanced between excitation and inhibition^34,35^. SOC argues that in systems that achieve global complexity through relatively simple local interactions, their dynamics form power law distributions which have since been identified in a variety of systems ranging from artificial neural networks to how tectonic plates interact to form earthquakes, and the interactions involved in COVID-19 outbreaks^36–38^.

SOC may engender several benefits to neural systems. Modeling has demonstrated that being at a critical point optimizes the information processing and propagation capacities of a system^34^. Furthermore, being at a critical point yields a balance between the competing demands of flexibility to adapt to changes in the environment while maintaining stable representations of learned and predictable behavior^39^. Notably, SOC enables more efficient optimization of artificial neural networks than many other processes that are widely used^40^. One caveat of this interpretation however is that while SOC is a compelling theory that could conceivably explain many of our findings and, more broadly, real world cognitive and neural behavior, there are alternative ways to generate power laws such as long-range statistical processes, successive fractionation, and combinations of exponentials^41,42^. In general, the dynamics we found could stem from a variety of causes ranging from neuroanatomy, endogenous physiological variation, environmental variation, behavioral trends, the behaviors of others, and a host of other factors.

Studying brain dynamics at this scale enables the study of cognitive and physiological processes inaccessible on shorter timescales. Someone’s attention, mood, and arousal oftentimes fluctuate on the order of hours-to-days. Physiological changes, such as dynamics related to circadian rhythms, hormones, and gene expression do the same. Recent technological advances providing the ability to record both neural activity^43^ and physiological biomarkers^44^ in an animal’s home environment can provide a fine-grained view into the cell-to-circuit neural behavior underlying cognitive and physiological fluctuations over hours-to-days. Clinically, many neuropathological states evolve and fluctuate over hours-to-days-to-years. In humans, chronic and continuous neural recordings that are performed as standard of care for certain patient populations (including fully natural and deployable recordings in patients with certain deep-brain stimulation systems^45^) can provide the opportunity to study real-world neural behavior on this timescale, both to understand basic neural behavior (as in this study) and to better understand their pathology. Stable and deployable wearable technologies for non-invasive neural recordings in real-world setting are also starting to be developed^46^. To help facilitate the development of these types of studies, we have made our analysis code available at https://github.com/MNobodyWang/WeekLongBrain.

Taken together, these results suggest that the functional flexibility and adaptiveness of our brains arise from unpredictable and chaotic-like trajectories that appear to explore many possible brain states before stabilizing into a steady-state solution, which is an emergent property of combining stable and consistent local brain dynamics^47^. The power laws of the overall statistics of these transitions suggest that this emergent phenomenon relates to self-organized critical processes in the brain.

## Methods

### Subjects

**Figure 1.**
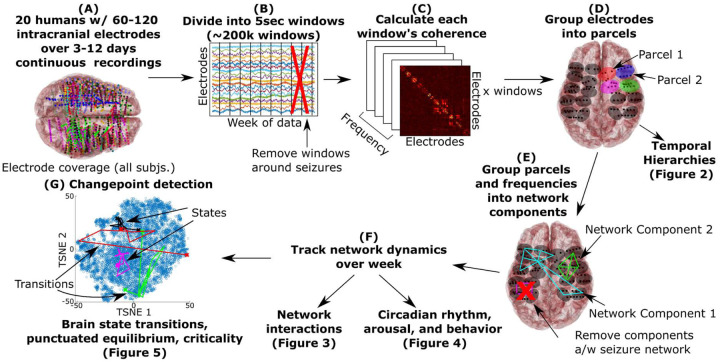
(A) We took between 3–12 days of continuous neural recordings from twenty participants and (B) split it into five-second-long non-overlapping windows, removing windows around seizure activity and filtering/regressing out artifacts. (C) We generated a functional connectome for each window as the coherence between all pairs of electrodes and removed additional artifacts by regression and independent component analysis. (D) We grouped electrodes with high coherence to each other over the week into parcels that tended to be anatomically close together (e.g. functional parcellation of cortical recordings). The parcel dynamics fell into temporal hierarchies that followed anatomical trends (Figure 2). (E) Parcels and frequencies that covaried were grouped into network components using a robust Principal Components Analysis. (F) The dynamics of these networks showed consistent pair-wise interactions (Figure 3) and relations to circadian rhythm, arousal, and behavior (Figure 4). (G) Overall brain state dynamics were assessed by finding transition points in the overall mixture of all networks’ evolution over time using change point detection. The mixture of all brain networks would fall into stable states punctuated by transitions that were complex, unique, and showed distributions consistent with self-organized criticality (Figures 5).

Twenty participants (nine males, 11 females; mean age 40 years with a standard deviation of 12 years) had intracranial surface or depth electrodes implanted for the treatment of pharmacologically intractable epilepsy (Figure 1A). All participants gave informed consent to participate under research protocols approved by the University of Pittsburgh Institutional Review Board. Depth electrodes were produced by Ad-Tech Medical and PMT and were 0.86 and 0.8 mm in diameter, respectively. Grid electrodes were produced by PMT and were 4 mm in diameter. Sixteen participants had depth electrodes only, three had grids only, one had a combination of both.

### Analysis overview

In summary, we collected and preprocessed lengthy intracranial recordings which we divided into five second windows (Figure 1A–B), calculated the functional partial connectome of each window via all-to-all electrode coherence (Figure 1C), grouped electrodes into tightly connected parcels which we analyzed (Figure 1D), grouped parcels and frequencies into functional network components using Principal Components Analysis (Figure 1E), and then studied the overall mixture of all functional networks (e.g. all principal components, Figure 1F). Artifacts were removed at multiple points in the analysis. Specifically, a comb filter was applied to remove line noise, an hour before, during, and after all seizures were removed to eliminate ictal and peri-ictal activity, spatial regression was used to remove local and global artifacts (such as motion, respiratory, and cardiac artifacts that tend to be similar in neighboring electrodes), ICA was used to remove large spike artifacts that sometimes occur due to disturbing the cables or connections, and epileptogenic areas and activity that correlated with the activity in these regions was removed to eliminate interictal activity or other pathological activity.

### Intracranial EEG data collection

Electrodes were localized via postoperative MRI or CT scans coregistered to the preoperative MRI using Brainstorm^48^. Surface electrodes were projected to the nearest point on the preoperative cortical surface automatically parcellated via Freesurfer to correct for brainshift^49,50^. Electrode coordinates were then coregistered via surface-based transformations to the fsaverage template using Freesurfer cortical reconstructions.

Intracranial electroencephalography data was collected using the Natus system at 1kHz. Notch filters at 60/120/180Hz were applied with a subsequent bandpass filter from 0.2 to 115Hz. The spatial autocorrelation between an electrode and all electrodes within 2cm of it was then measured and regressed out to remove both local and global artifacts, including artifacts due to motion and current spread due to volume conduction.

An hour before and an hour after all seizures, electrographic or clinical, were removed before calculating coherence (Figure 1B). A board-certified neurologist identified the seizure network in all but two participants, with those two participants having no recorded electrographic or clinical seizures during their stay in the hospital.

The data was then separated into five second windows (Figure 1B) with coherence computed over each window between all pairs of electrodes over five frequency bands: theta (4–8Hz), alpha (8–12Hz), low beta (14–20Hz), high beta (20–30Hz), and gamma (30–70Hz). In summary, this generated five connectome structures every five seconds (Figure 1C). This was performed using scipy coherence function under default settings as of version 1.9.3.

Independent component analysis was then applied, and components were visually inspected for any artifacts which were then removed. The main criteria for removal were independent components that possessed time course activations that were clearly non-neurological (such as step-functions or near Dirac deltas).

### Parcellation (Figure 1D)

For each participant, we parcellated their electrodes into groups of tightly coherent electrodes (Figure 1D). We utilized the Leiden algorithm to identify a single regional atlas that optimized graph modularity over the entire week-long period across all five frequency bands^51^. Modularity (Equation 1) was calculated separately over each network from every five-second window with the Leiden algorithm optimizing the average modularity across all windows and frequencies. This generated on average 10–15 parcels for each participant.

$$\text{ modularity }=\sum_{b\in \left\{\theta ,\alpha ,\beta_{l},\beta_{u},\gamma \right\}}\sum_{t}\sum_{i,j}\left[A_{i,j}^{b,t}-\frac{k_{i}^{b,t}k_{j}^{b,t}}{2m^{b,t}}\right]\delta \left(c_{i},c_{j}\right)$$

Equation 1: Modularity metric that Leiden algorithm optimizes. $A_{i,j}^{b,t}$ refers to the weighted connectivity (coherence) between electrodes *i* and *j* at time window *t* and frequency band *b*. $k_{i}^{b,t}$ is the degree of electrode *i* and *m*^*b,t*^ is the sum total of all connections at that time and frequency. *δ(c*_*i*_,*c*_*j*_) is an identity function as to whether electrodes *i* and *j* are in the same parcel. The Leiden algorithm finds the parcellation assignment of each electrode that optimizes modularity over all time windows and frequency bands.

To assess the stability of which electrodes would be grouped into which parcels, we separated the data into six-hour non-overlapping segments (between 18–80 segments per participant) and found the optimal community structure for each segment. We quantified the similarity between each segment’s parcel definitions using the Rand Index^52^ (percentage of electrode pairs that were parcellated equivalently under the two parcel definitions) which almost universally returned values greater than 0.9 as illustrated in Supplementary Figure S2, indicating that the overall parcellation was well-preserved over time, motivating our decision to use the same parcel structure over the entire work for interpretability.

We further removed any activity from the remaining non-seizure-related parcels that correlated with activity from parcels within the participants’ seizure onset zones outside of ictal periods to ensure that any potential pathological activity was removed of our analysis.

### Autocorrelation stability (Figure 2)

**Figure 2.**
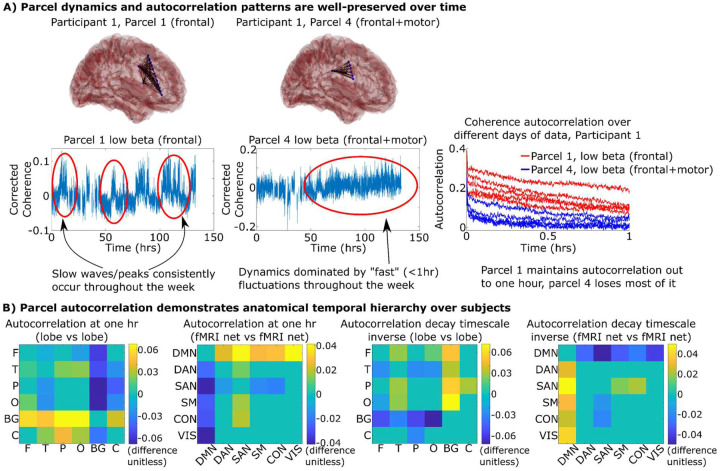
A) The coherence within two parcels from a representative participant (left) and the autocorrelations of those regions calculated on each day separately (right). Autocorrelation curves of the same color represent the autocorrelation of that parcel on different days of data. Each parcel’s coherence displays a “unique” temporal signature that is conserved over days that is reflected by stability in their autocorrelation curves. Breaks/skips in data represent windows removed due to seizure activity. Comparisons between all parcels for each participant are shown in Supplementary Figure S3. B) Pairwise comparisons between the autocorrelation of parcels (averaged over frequency and participants) that belong to one lobe (F: frontal, T: temporal, P: parietal, O: occipital, BG: basal ganglia, C: cingulate)/fMRI resting network versus another. Cell values indicate the difference in autocorrelation (y axis minus x axis) between two regions or networks found using linear mixed effect models. Autocorrelation decay timescale inverse is an indicator on how sharply the autocorrelation curve decayed with lower values indicating less autocorrelation decay at high timescales. Non-zero cells indicate statistically significant differences post multiple comparisons correction (p<0.05).

We tested whether the autocorrelation of each parcel’s coherence would show relatively consistent patterns of “fastness” or “slowness” throughout the week (Figure 2). We split the week-long time course for each participant into six-hour non-overlapping segments. After removing parcels associated with the seizure network, we then took the average coherence between electrodes within a single parcel for a single frequency band and then calculated its autocorrelation up to one hour. We fit this autocorrelation curve to a power law $\left(\text{autocorrelation }(t)={\text{AC}}_{1}\times {\text{time}}^{-{\text{AC}}_{2}}\right)$ to generate two timescale parameters: AC_1_ (autocorrelation strength) and AC_2_ (autocorrelation steepness) which described the autocorrelation of a single parcel at a single frequency at a single time segment. For a given frequency band, we took the timescale parameters across all parcels and time segments and grouped the parameters by which parcel they were measured in. We used Kruskal-Wallis ANOVA tests to show that in almost all participants and frequency bands, there were statistically significant differences between the group means, mostly with high effect sizes (η>0.12, Supplementary Figure S3).

We tested whether parcels from different anatomical regions tended to have reliable differences in their autocorrelation across participants using linear mixed effect models^53^. We assigned each parcel to a lobe and canonical fMRI network based on its largest overlap. Parcels with less than 60% of their electrodes belonging to the same anatomical group were excluded for this analysis. For each parcel, we calculated the autocorrelation of its average intra-parcel coherence for a given frequency over the entire week out to one hour and calculated AC1 and AC2 as described above. We then averaged both parameters across all frequency bands.

We then chose a single pair of lobes or fMRI networks (such as frontal vs temporal) and selected all the parcels across our participants that fell into one of those two anatomical groups. We used MATLAB’s fitlme (linear mixed effect model) to model each parcels autocorrelation parameters with both the participant and the anatomical group as fixed-effects, allowing us to determine whether one anatomical group had a reliably higher autocorrelation parameter than the other. We repeated this for all possible pairs of lobes/fMRI networks and used Bonferroni multiple comparisons correction to identify pairs with significant differences (Figure 2B).

### Robust principal components analysis (Figure 1E)

We found that many parcels tended to be highly colinear with each other as shown in Figure S17. To reduce dimensionality, we used a modified PCA protocol. While the results in this manuscript still hold statistical significance without this dimensionality reduction as shown in Supplementary Figures S12 to S16, parcel pair-wise interactions are less interpretable due to a high degree of covariance between them, and some classifier performance degrades slightly (likely because some noise is removed by only including high variance principal components, we use a 90% variance explained cutoff).

We grouped parcels and frequencies that tended to covary together using random sample consensus PCA (RANSAC-PCA) on the parcel coherences (Figure 1E). By taking the average intra-parcel coherence during each time window and frequency, we formed a (number of parcels × 5 frequency bands) by (number of time windows) 2D matrix which we then reduced to a (number of components) by (number of time windows) matrix using a modified PCA protocol. This identifies parcels and frequencies that tend to strongly covary together that we could easily interpret as a single network component feature that captures cross-frequency relationships while also reducing the dimensionality of the original dataset to simplify further analyses.

Our modified PCA protocol uses random sample consensus to avoid PCA’s susceptibility to noisy outliers by attempting to exclude outliers by taking multiple small subsamples of the data and selecting one with the fewest number of outliers to train the model^18,54^. We generated 1000 subsamples where in each subsample, we selected six 30-minute segments of data from each day. This ensures that the PCA is robust to rare outliers and that the PCA produces principle components that are stable across days within a participant. Outliers were defined by calculating the Mahalanobis distance between each time window’s feature vector and each subsample’s distribution. In each participant, we found that these distances would take on clear bifurcations between relatively small distances and short “spikes” of extremely high distances (more than three standard deviations) away from the mean that typically lasted for a few minutes. We manually drew a cutoff for each participant that was approximately half the average Mahalanobis distance of these spikes. For each subsample, we calculated the number of outliers within the subsample, and calculated PCA over the subsample with the fewest outliers. We utilized enough PCs to capture 90% of the variance in the dataset, generally resulting in 12–24 networks/PCs per participant.

The network component activation of a principal component was defined as the projection of the parcel coherences onto the principal component weights. We repeated the same autocorrelation stability analysis described above on each network component’s activation (Supplemental Figure S5).

### Seizure network removal

**Figure 3.**
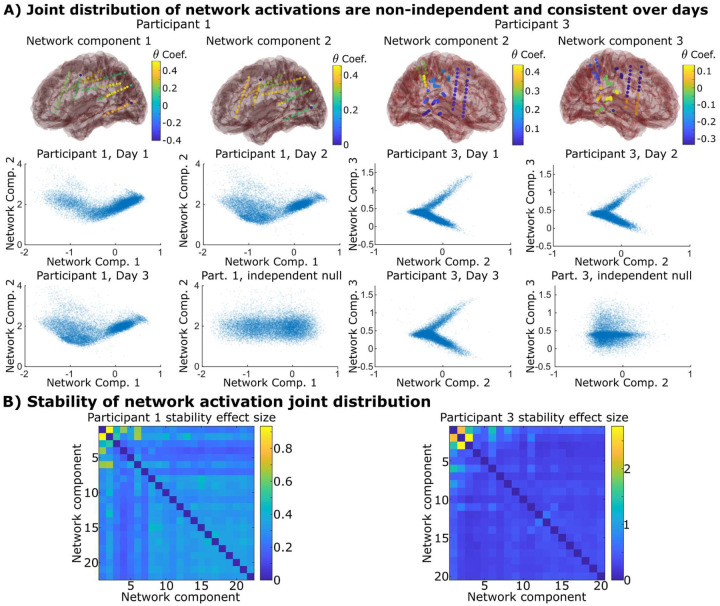
A) A pair of networks/principal components from two participants that showed non-independent distributions that are conserved over different days of data. The null distribution if the network components were independent of one another are also shown for comparison. B) The distance between the joint distribution of each pair of networks/principal components compared to the null distribution where they are independent of each other. Non-zero effect sizes represent statistically significant distances as determined via permutation testing. We find several pairs that demonstrate such relationships, most notably the lower network components that capture most of the variance in the dataset. Supplementary Figure S6 shows that all twenty participants possessed several such pairs.

**Figure 4.**
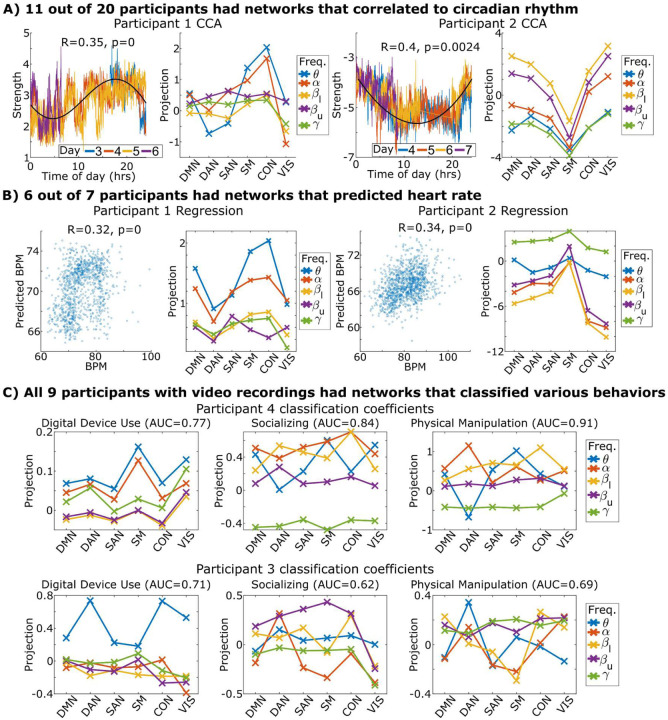
A) We linked network component mixtures to circadian rhythm by training canonical correlation analysis on one half of the week and then testing on the other. The network mixture activations during testing are shown on the left plotted against time with the black line indicating a theoretical circadian rhythm. Skips in data are removals due to seizures or disconnected hardware. The identified mixture’s anatomical and frequency coverage are shown projected onto the canonical fMRI networks. B) Network components were linked to heart rate by training linear regressors on one half of the week and testing on the remaining half. Test predictions are plotted against heart rate along with their anatomical and frequency coverage. C) Logistic classifiers identified network components that reliably detected whether the participant was watching a digital screen, socializing with another human, or physically interacting and manipulating a held object. The algorithm was trained over one randomly selected day and tested on another. The classifier’s anatomical and frequency coverage is shown on the right. Area under the curve (AUC), a classifier performance metric, is shown in the figure title. All participants for these analyses are shown in Supplementary Figures S7–S9.

When analyzing parcel dynamics (Figure 2), we excluded all parcels with electrodes part of the seizure onset zone and early propagation as defined by a board-certified neurologist. For network component dynamics (Figure 3 onwards), we first re-added these seizure-related areas before grouping parcels and frequencies into network components through robust principal component analysis. We then removed any network components that were associated with the seizure network before analyzing their dynamics. More specifically, we calculated the dot product similarity between the absolute value of a principal component vector (normalized to a magnitude of one) and a binary vector that marked all electrodes that were part of the seizure network (also normalized to one). The similarity between these two vectors indicated how anatomically similar the driving factors of a principal component and the seizure network were to each other. A null distribution for this similarity was formed by randomly permuting the principal component vectors, and all principal component vectors that showed statistically significant similarity to the seizure network (p<0.05) were removed from all further analyses.

### Network components show non-independent relationships that are well-preserved over days (Figure 3)

We tested whether network components had reliable interactions with each other by examining their joint distribution stability. For each pair of network components in a participant, we divided the total range of each component’s activation into 1000 discrete bins to generate a 1000 × 1000 grid covering the space of both components’ activation. We calculated the empirical joint distribution of the network component pair over these bins for each day separately. We calculated the Bhattacharyaa distance^55^ between each day’s distribution to each other as well as the distance to the expected joint distribution if each network were independent but possessed the same marginal distribution (which we denote the expected independent distribution). The “effect size” metric shown in Figure 3 is the average distance between the real distributions on different days divided by the distance to the expected independent distribution. Using permutation testing (10k trials), we established a null distribution for effect size if the networks were in fact independent by randomly drawing however many days of samples we had from the expected independent distribution and calculating the effect size over these draws. Statistical significance was then corrected for multiple comparisons across all possible pairs of joint network distributions using Bonferroni correction.

### Network component activation is tied to circadian rhythm, heart rate, and behavior (Figure 4)

We tested whether we could identify combinations of network components that were associated with neurophysiologically relevant markers. More specifically, we looked at circadian rhythm, heart rate, and behavior.

Canonical correlation analysis (CCA) was used to identify a mixture of network components that matched a circadian sinusoid with a period of 24 hours. The circadian sinusoid was defined as a1*cos(t/24hrs)+ a2*sin(t/24hrs) where a1 and a2 are constants learned by CCA. CCA simultaneously tried to find a linear combination/weighting of network component activations to fit to this sinusoid.

The model was trained over the first half of the week and then tested on the second half through Pearson correlation (out of sample validation of correlation). The Pearson R of the fit on the test dataset was calculated and then compared to a null distribution of R that was formed via permutation tests that temporally shifted each day’s network component activity forward or backwards by a uniform random number ranging from 0–24 hours. This preserves the autocorrelation of the neural signals while eliminating any consistent circadian-like pattern across days.

Heart rate was assessed using collected EKG signals that were processed using heartpy^56^. The instantaneous heart rate for any window was the average heart rate for a 30-second period centered on the window. L1-regularized regression was trained on the first half of the week to identify a mixture of networks that predicted heart rate using sklearn’s implementation (out of sample validation of regression). Hyper parameterization was optimized on the training set using ten-fold cross-validation. The quality of the fit was assessed on the remaining half via Pearson correlation with a null distribution formed using the same permutation tests used for circadian rhythm to preserve both the autocorrelational properties of the heart rate and neural signals.

Video and audio recordings of the participants from two separate angles were used to assess the participant’s behavior over two randomly selected days via manual annotation. Digital device usage was defined as looking at any digital screen, such as a smartphone or laptop. Socialization was defined as verbal communication with another human being, or in one case a canine companion, either in person or over the phone. Physical manipulation was defined as actively grasping and interacting with any physical object or person. These three behaviors were not mutually exclusive with one another.

Windows with the desired behavior were manually annotated on two separate days of data. L1-regularized logistic classification was used to identify a mixture of network components that classified each behavior independently using one day for training and the other for testing using sklearn’s implementation (out of sample validation of classification). Hyper parameterization was optimized on the training set using ten-fold cross-validation. The area-under-curve of the receiver-operator-curve of each network’s ability to classify the desired behavior was calculated.

### Transitions in the overall brain state fall into a punctuated equilibrium (Figure 5)

We examined how the overall brain state (the status of all recorded network components in the brain) would change over time by dividing the week into “transitions”, periods when the brain was rapidly reconfiguring itself, and “states”, periods of time where the brain’s functional connectome appeared to be relatively stable.

**Figure 5.**
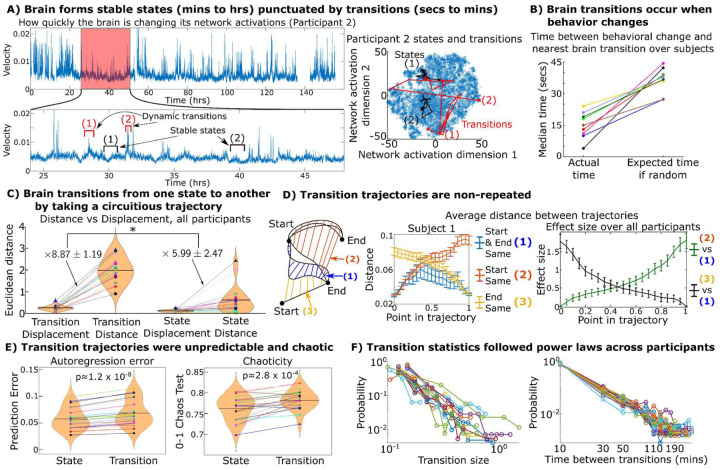
A (left) Overall change in network activations between consecutive time windows for one participant (Euclidean distance between the vector of all network activations from one time window to the next). Two segments of states and transitions are marked. A (right) T-distributed stochastic neighbor embedding visualized the week-long time course of network activations. (B) We detected when the brain state was transitioning using changepoint detection and asked whether these times corresponded to when the participants’ behavior changed. We compared the median time between behavioral changepoints and the nearest brain transition to the expected time if random and found that neural and behavioral changepoints occurred together (p=1e-4 by paired t-test). (C) We tested whether brain transitions went directly from one state to another or whether they took indirect, circuitous routes. We plotted the average total distance (sum of velocity) traveled during transitions and stable states for all twenty participants versus their net displacement (distance between start and end state). Net distances for all participants were several times larger than the net displacement, indicating that transition trajectories were lengthy and indirect. The ratio between distance and displacement were higher for transitions than states (paired t-test). D (top) Average distance between pairs of transition trajectories as a function of what proportion of the trajectory was complete. Trajectory pairs are grouped into three categories: transitions with similar starting and ending points (1, blue) vs similar starting points only (2, red) vs similar ending points only (3, yellow). These distances are shown for one participant in the middle with all participants are shown in Supplementary Figure S10. The effect size of the differences between these distances over all participants is shown on the right. (E) We used autoregression to demonstrate that the predictability of brain dynamics decreases during transitions. We used a 0–1 chaos test to demonstrate that the chaoticity of brain dynamics rises during transitions. (F) The distribution of the size (net displacement) of each transition and the time between them is shown for all participants in log-log form. Both distributions formed power laws (linear on log-log axes) consistently across participants.

**Figure 6:**
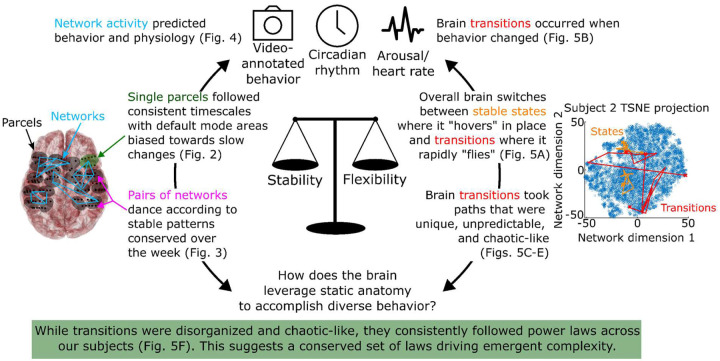
Summary of results presented in this manuscript and of the overall dichotomy between a brain made of simple, stable “parts” that is still capable of generating complex group-level behavior as it reacts to an ever-changing environment over long time periods.

In Figure 5A and Supplementary Figure S18, we provide evidence that the brain falls into states and transitions by examining the “velocity” of the brain. Velocity was defined as how much the brain’s state changed between one five-second window and the next. More specifically we took the vector of all network activations of each window (the parcel coherences projected into the network PCA space) and calculated the Euclidean distance between the network activation vector of one window and the next. Speed is technically a more appropriate term than velocity from a kinematics perspective. However, we avoided the term speed due to its connotation with cognitive processing speed which is unrelated to this analysis.

We calculated the distribution of the time between windows that fell into the top 1% of velocity and compared that distribution to Poisson distributions with λ=0.01. The Poisson distribution captures what the expected time between high-speed windows would be in a memoryless process (non-autocorrelated speed). By demonstrating that windows with high speed tended to cluster next to each other temporally, we found that there were specific periods of time when the brain is quickly changing and times when the brain is relatively static. We also tested this between windows falling within the top 10% of velocity (λ=0.1) and found the same result.

In Figure 5B, we evaluated whether state transitions were linked to behavioral changepoints by marking out times when the participant’s behavior changed in any of the three categories. We calculated the median time between this changepoint and the nearest state transition (zero if the changepoint occurred during a transition). We calculated an expected value for this metric assuming no relationship by temporally shifting the behavioral changepoints forward or backwards by a uniform random number ranging from 0–24 hours, calculating the median time difference, and then averaging over 1000 trials. We tested whether the real time difference between behavior changes and state changes was consistently smaller than the expected time difference using a non-parametric t-test across participants (paired t-test).

Next, we analyzed properties of these transitions (Figures 5C–E). We defined transitions in two different ways to ensure our conclusions were not overly method dependent. The transitions described in the main text figures were identified using binary segmentation change point detection on the overall brain state. The transition trajectory was defined as the period around a change point that possessed above-average velocity (the Euclidean distance between the vector of all network activations from one time point to another). If the trajectories associated with two neighboring change points overlapped, the change points were “merged” into one.

We replicated Figures 5C&D with a different trajectory definition in Supplementary Figure S11. We calculated the velocity over the week and took the moving average of it over a 30 second window and defined trajectories as periods of time when this smoothed speed reached the top 20% quantile over the week. We replicated Figure 5E in Supplementary Figure S11 where we found the main relationships highlighted in these figures still held true.

In Figure 5C, we examined the “distance” and “displacement” of these transitions. During a transition, we calculated the speed between each window. The distance was the sum of those speeds. The displacement was the distance between the first window of the transition and the last. This comparison allowed us to determine how direct the transitions were because in a “straight line” transition, distance would equal displacement, but if distance is much greater than displacement, then the transition is more circuitous.

In Figure 5D (top), we examined the distance between the paths traversed by different transition trajectories. We calculated the distance between the start points of all trajectories in a participant and the distance between their end points. Two trajectories were considered to have the same starting or ending point if the distance between the points fell into the bottom 10% of trajectory pairs. We grouped trajectories into three groups: trajectories with the same starting and ending point, trajectories with the same starting point only, and trajectories with the same ending point only. We calculated the average distance between trajectories that fell into each group as a function of how much of the trajectory has been completed. More specifically, we used linear interpolation to determine what was the brain state 5%, 10%, 15%, 20%, …, 95% of the way into each trajectory. We calculated the distance between brain states of the same percentage in each of the three groups. Figure 5D shows the distribution of these distances for a single participant along with the effect size of the difference between these distances across all participants. For effect size, for each subject individually, we calculated the Cohen’s d between the distances between trajectories that start and end the same to the distances between trajectories that start the same but end differently or trajectories that end the same but start differently. This measures the number of standard deviations that separate the distributions in the trajectory categories at different points along the trajectory. We then calculated the standard error of these Cohen’s d across all 20 subjects. The average Cohen’s d and these standard errors are shown in Figure 5D.

In Figure 5E, we studied whether these transitions influenced the autoregressive prediction error and chaoticity of the brain dynamics. Our autoregressive model was vector autoregression with the number of previous time-steps being selected using Bayesian Information Criterion. These models were trained and evaluated using cross-validation by “holding out” one day of data and training on the remainder of the week. The predictive error was the average mean-squared error on the held-out day during either transition or state dynamics. Chaoticity was defined using the 0–1 chaos test using the protocol described in^26^ and was calculated over non-overlapping ten minute segments. In summary, we calculated the chaoticity of each network component independently over each time segment. The chaoticity of the overall neural dynamics for a given time segment was defined as the median chaoticity of all network components. Segments with a transition were compared to segments without one.

(2.1) p(n+1)=p(n)+ϕ(n)coscn

(2.2) q(n+1)=q(n)+ϕ(n)sincn

(2.3) $$M(n)=\underset{n\to \infty }{\text{lim}}\frac{1}{N}\sum_{j=1}^{N}\left({\left[p(j+n)-p(j)\right]}^{2}+{\left[q(j+n)-q(j)\right]}^{2}\right)$$

(2.4) $$K_{c}=\underset{n\to \infty }{\text{lim}}\frac{\text{log}\;M(n)}{\text{log}\;n}$$

Equation 2: Chaos 0–1 test protocol. Define *φ(n)* as the network component activation of interest at time window *n* for a given ten-minute segment. This is used to “drive” the dynamical system described in Equations 2.1 and 2.2 where *c* is a randomly chosen “resonance” parameter between 0 and π that remains constant during a single “iteration” of this process. *M(n)* is evaluated up to an *n* of approximately *N*/10 where N is the number of time windows in the ten-minute segment. *K*_*c*_ is estimated by fitting a straight line between the numerator and denominator of Equation 2.4 and represents the chaoticity of a single iteration. *c* is redrawn 1000 times and the median *K*_*c*_ is defined as the chaoticity of the network component over the ten-minute segment.

In Figure 5E, we analyzed the distribution of the transition size which we defined as the net displacement of a transition and the time-between transitions. We fit power law exponents to these distributions using MATLAB’s nlinfit function with power laws defined as a1*frequencŷ(-a2) where a1 and a2 are learned.

We tested whether these distributions came from power law distributions using two methods from^57^. First, we used Kolmogorov-Smirnov (KS) tests to test whether we failed to reject the null hypothesis that the distributions plausibly came from power laws. We fit power law distributions to each participant’s transition size and time between transitions distributions separately and calculated the KS distance between the experimental distributions and their theoretical power law ones. We formed a null distribution on these distances by drawing 1000 random samples from the theoretical power law distribution, fitting a power law distribution to those samples, and then calculating the KS distance between the sampled distribution and the fitted one. If these distances were consistently lower than the distance between the real distribution and its estimated power law one, then we reject the null and conclude that the distribution did not come from a power law. We found that 17/20 participants had transition size distributions that plausibly came from power law distributions (p>0.05), and 20/20 participants had time-between transition distributions that plausibly came from power law distributions.

We then used likelihood comparison tests to see whether the transition size and time-between distributions were more likely to have come from power law, exponential, or log-normal distributions. We calculated the log-likelihood that each participant’s distributions came from each of the three categories. We used a Wilcoxon signed-rank test to test whether the log-likelihood of power law distributions were higher than exponential and log-normal distributions across participants. We found that power law distributions were more likely than exponential (p=0.007 for transition size and p=4.8e-5 for time-between) and more likely than log-normal (p=0.03 for transition size and p=4.8e-5 for time-between).

## Data Availability

The data that support the findings of this study are available from the corresponding authors upon request.

## Footnotes

* To ensure that the rest of the results were robust to this PCA procedure, we examined whether the results held without using PCA for dimensionality reduction. The results in Supplementary Figures S12–S16 show that our results remain statistically significant, though the effect sizes declined likely because the power of our analysis increases by grouping together covarying parcel activity into functional network components and by removing noisy, low variance PCs.

† Technically, speed is a more appropriate term than velocity from a kinematics perspective. However, we avoided the term speed due to its connotation with cognitive processing speed which is entirely unrelated to this analysis.
