# A week in the life of the human brain: stable states punctuated by chaotic transitions

**DOI:** 10.21203/rs.3.rs-2752903/v2

**Published:** 2023-10-02

**Authors:** Maxwell B. Wang, Max G’Sell, James F. Castellano, R. Mark Richardson, Avniel Singh Ghuman

**Affiliations:** 1 Neuroscience Institute, Carnegie Mellon University, Pittsburgh, PA, USA; 2 Machine Learning Department, Carnegie Mellon University, Pittsburgh, PA, USA; 3 Department of Neurological Surgery, University of Pittsburgh, Pittsburgh, PA, USA; 4 Medical Scientist Training Program, University of Pittsburgh and Carnegie Mellon University, Pittsburgh, PA, USA; 5 Department of Statistics and Data Science, Carnegie Mellon University, Pittsburgh, PA, USA; 6 Department of Neurology, University of Pittsburgh, Pittsburgh, PA, USA; 7 Department of Neurosurgery, Massachusetts General Hospital, Boston, MA, USA; 8 Harvard Medical School, Boston, MA, USA; 9 Center for the Neural Basis of Cognition, University of Pittsburgh and Carnegie Mellon University, Pittsburgh, PA, USA; 10 Center for Neuroscience at the University of Pittsburgh, Pittsburgh, PA, USA

## Abstract

Many important neurocognitive states, such as ones related to performing natural activities and fluctuations of arousal, shift over minutes-to-days in the real-world. We analyzed 3–12 days of continuous intracranial recordings in twenty participants that freely socialized, used digital devices, slept, etc. to understand how neural dynamics form and change with behavior. Brain networks formed stable states that were predictive of both behavior and physiology. Behavior changes were associated with bursts of rapid neural fluctuations where brain networks chaotically explored many configurations before settling into new states. These trajectories traversed an hourglass-like structure, with awake and sleep at opposite ends, and an attractor state represented by default mode network activation in between. These findings illustrate ways our brains balance stability and flexibility to produce real-world behavior.

## One-Sentence Summary

During a week of intracranial recordings, the brain forms a punctuated equilibrium of stable states and chaotic transitions.

Whether we are fatigued from attending a teleconference call or eager to read a book, whether we feel vibrant and ready to start our day or weary and winding down to sleep, many neurocognitive processes in our lives slowly fluctuate over minutes to hours. Yet, most of our understanding of human brain activity comes either from well-controlled experiments, studying reactions to carefully chosen stimuli over milliseconds to seconds, or examining spontaneous neural activity from subjects “resting” inside a neuroimaging machine. A few studies have analyzed brain state dynamics over minutes in a single sitting or repeatedly sampled a few minutes per day spread out over days to months using functional neuroimaging (1–8). A small number of studies took similar snapshot approaches in real-world settings, such as tracking depression phenotypes (9, 10) or classifying small windows of a few specific behaviors (11, 12).

As a result, it is still unclear how our brain activity continuously changes over timescales of minutes-to-hours-to-days, particularly during natural behaviors that depart from the boundaries of conventional experimental paradigms. Here we asked several questions regarding long timescale brain dynamics: What neural states emerge during natural behavior when recorded continuously over a week using intracranial monitoring? How does the brain transition between these states? Do these states and transitions follow a consistent organization with respect to behavior, physiology, and anatomy across people?

We used intracranial neural recordings (80–126 electrodes implanted per participant) in twenty neurosurgical participants undergoing evaluation for epilepsy surgery for between 75 to 283 hours (near-continuous recordings across approximately 3–12 days). During this time, participants were confined to the hospital but would freely socialize with friends, family, and staff, interact with digital devices, sleep, watch TV, and perform other volitional natural behaviors while under simultaneous neural and video monitoring. We started by examining the basic dynamics of different areas of the brain, finding that they would change in consistent manners, albeit oftentimes in a complex and nonlinear fashion. To probe this nonlinearity, we used self-supervised deep recurrent neural networks and Koopman operators to learn a “state-space” of the brain’s dynamics – a geometric representation of the brain’s activity where changing patterns of brain network activations are reflected as movement in this state-space. This state-space allows us to query brain networks’ overall organization, relationships to behavior, and primary dynamical driving forces.

## Functional parcels showed consistent fluctuations over days and their dynamics displayed consistent anatomic trends.

**Fig. 1.**
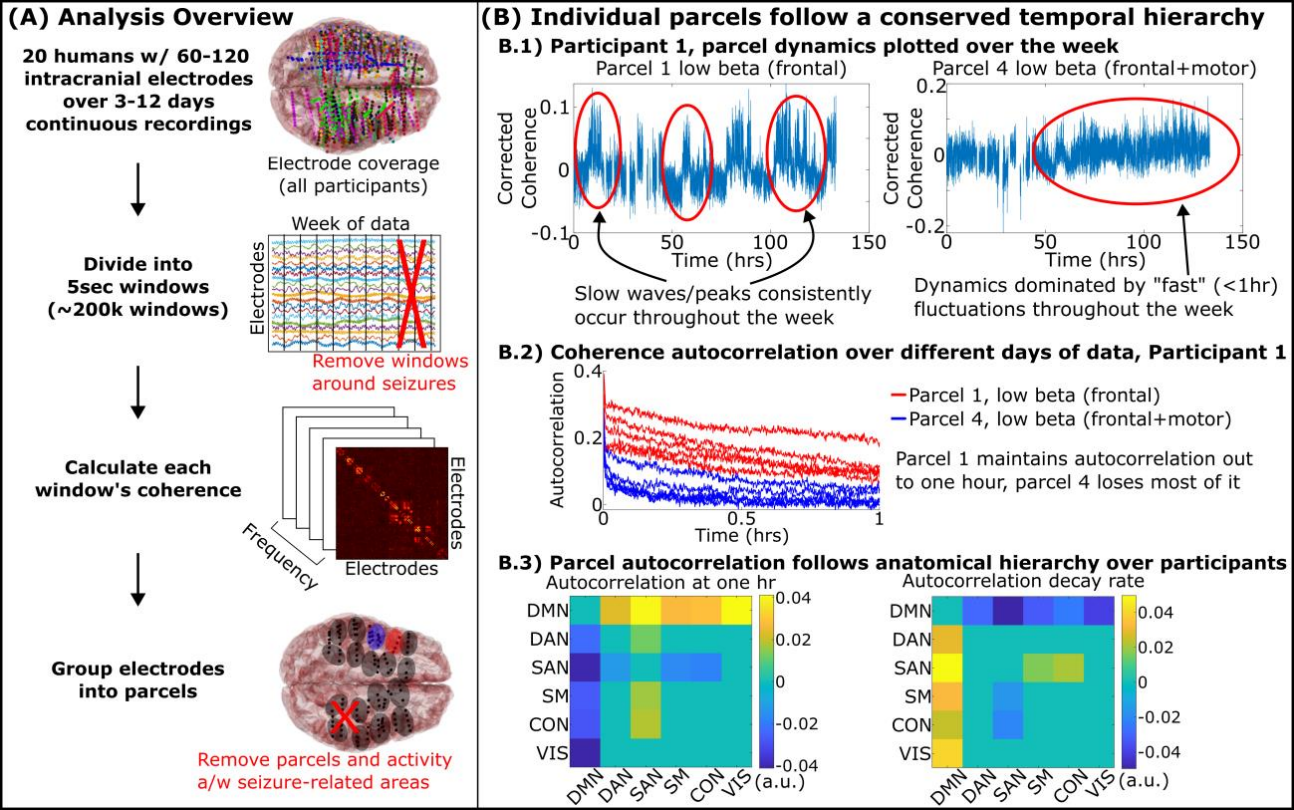
Parcels of the brain followed stable rhythms conserved throughout the week that followed an anatomical hierarchy. A) 3–12 days of continuous recordings from twenty participants were split into five-second-long windows, removing windows around seizure activity and artifact removal. We calculated the coherence between all pairs of electrodes and grouped electrodes with high coherence into anatomically compact parcels. B.1) Coherence within two parcels from a representative participant. B.2) Parcels display unique, stable timecales reflected by their autocorrelation stability over different days of data (all participants shown in Supplementary Figure S3). B.3) Timescale of rhythms between parcels belonging to one fMRI resting network versus another. Cell values indicate the difference in autocorrelation parameter across participants (y axis versus x axis) with positive cells indicating the network indicated by its row has a larger parameter than the one indicated by its column. Non-zero cells indicate statistically significant differences post multiple comparisons by mixed effects model. Methods described in Supplementary Section M6.

After removing an hour before and after ictal (seizure) events as determined by the clinical team, we calculated the coherence between all pairs of electrodes in each participant every five seconds over five frequencies: theta (θ: 4–8Hz), alpha (α: 8–12Hz), low beta (β_l_: 14–20Hz), high beta (β_u_: 20–30Hz), and gamma (γ: 30–70Hz) (13). Electrodes were parcellated into tightly connected, anatomically compact groups of electrodes, each a “parcel” of the brain (Figure 1A). After removing parcels and activity associated to the participants’ seizure onset zones, we plotted the coherence of each parcel over the week (Figure 1B-top). Fluctuations of these coherences showed characteristic temporal scales that were repeated over different hours and days of data. We quantified this stability by how slowly each parcel’s autocorrelation decayed (timescale). Timescale differences between parcels were stable over time, indicating that parcels that fluctuated faster or slower would remain so throughout the week (Supplementary Figure S3).

Differences between parcels were quantified over all twenty participants by grouping parcels according to which of six canonical fMRI networks they fell in (“default mode”, “dorsal attention”, “salience”, “somatomotor”, “control”, and “visual” as defined in (14)). Parcels in the default mode consistently showed higher autocorrelation magnitude and longer decay timescales across our participants, whereas parcels of the salience network showed shorter timescales (Figure 1B-bottom). These findings demonstrate a temporal hierarchy separating “fast” and “slow” regions of the brain. A temporal hierarchy, typically measured using autocorrelation, has been hypothesized in the brain with transmodal systems, such as the default mode network, slowly integrating data from faster unimodal regions over seconds to minutes depending on the task (1, 2, 15, 16). Our results extend these findings to minutes-to-hours in a real-world setting during natural behavior.

## Neural dynamics predicted physiology.

**Fig. 2.**
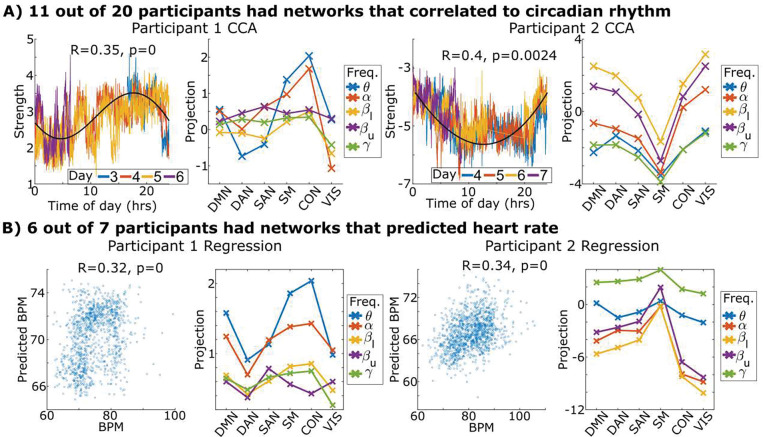
Brain networks predicted physiological markers. A) We linked networks (groups of parcels found using principal components analysis) to circadian rhythm by training canonical correlation analysis on one half of the week and then testing on the other. The network mixture activations during testing are shown on the left plotted against time with the black line indicating a theoretical circadian rhythm. Skips in data are removals due to seizures or disconnected hardware. The identified mixture’s anatomical and frequency coverage are shown projected onto the canonical fMRI networks. B) Networks were linked to heart rate by training linear regressors on one half of the week and testing on the remaining half. Test predictions are plotted against heart rate along with their anatomical and frequency coverage. All participants for these analyses are shown in Supplementary Figures S5 and S6. Methods described in Supplementary Section M9.

To assess the neurophysiological relevance of the above dynamics, we linked them to fluctuations in circadian rhythms and arousal. After grouping parcels into networks to reduce dimensionality in a data-driven fashion, we took the first half of the week for each participant and used canonical correlation analysis (CCA) (17) to identify networks that maximized correlation to time of day. We tested this group of networks on the second half of the week using permutation testing and found that 11 of the 20 participants had networks significantly linked with circadian rhythm (Figure 2A). Notably, six of the nine participants whose data lacked significant correlation to time of day had sleep disturbances such as nocturnal-awakening seizures or intentional clinical sleep deprivation, suggesting these participants had disrupted circadian rhythms.

Seven participants had sufficiently clean electrocardiogram (EKG) signals to track heart rate. Heart rate is strongly correlated with the degree of arousal (18) and is used here as a proxy for it. We used L1-regularized (19) regression over the first half of the week to identify a group of networks that predicted heart rate and tested this group on the remaining half (Figure 2B). Six of the seven participants had networks that were significantly associated with heart rate.

## Brain networks underwent bursts of rapid transitions that coincided with natural behavior shifts.

**Fig. 3.**
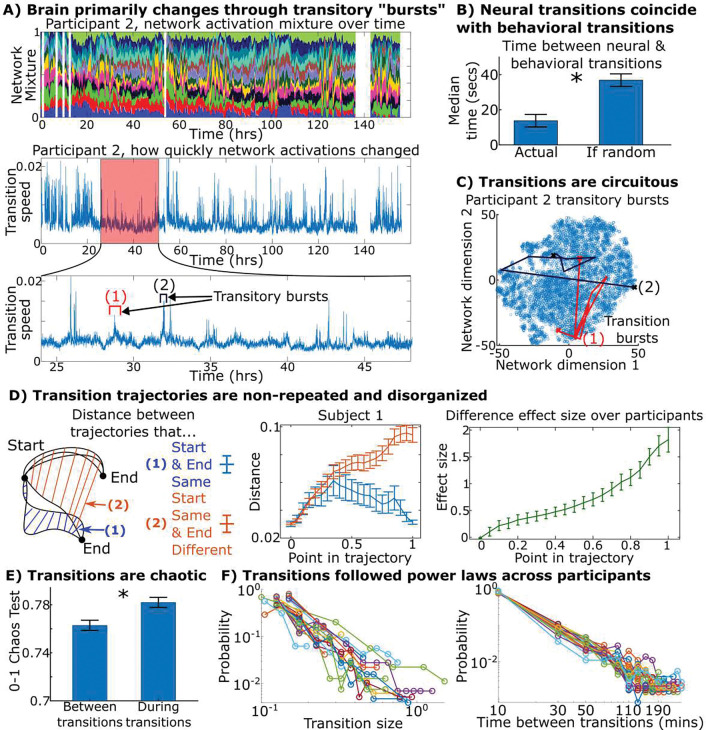
Neural dynamics undergo chaotic-like transitions when natural behavior shifts. (A-top) Network activations plotted over the week for one participant where each color represents the activity of a different network (sum of activity normalized to one for visualization purposes). (A-mid/bottom) How quickly the brain changed network activations every five seconds. The brain reorganized itself using “transition bursts” of high speed (Supplementary Figure S7 quantitatively demonstrates that transitions are “bursty” across all participants). (B) Average time across participants between neural and behavioral transitions compared to the expected time if no relation between the two. Neural and behavioral transitions tended to occur with each other (p=1e-4, paired t-test). (C) Two transitions visualized on a t-distributed stochastic neighbor embedding of the brain’s weeklong course, showing that transitions did not move directly between states but rather explored many interim states (quantified in Supplementary Figure S13 over participants). (D) We took transition bursts with the same starting and ending states and asked how similar they were as transitions progressed from start to end (1, blue). We compared this to transitions with the same starting but different ending ones (2, red). The Cohen’s d effect size on the difference between these two distances is shown on right. The first half of the transitions indicated little about the eventual destination, indicating that transitions in the brain did not take consistent paths from start to end. (E) 0–1 chaos test shows that the chaoticity of brain dynamics rises during transitions across participants (p=1e-3, paired t-test). (F) Distribution of transition size and the time between them for all participants in log-log form. Both distributions formed power laws (linear on log-log axes) across participants by Kolmogorov-Smirnov and likelihood tests. Details in Supplementary Section M10.

We next assessed overall brain network dynamics and the relationship between these dynamics and natural behavior. The top of Figure 3A shows the brain network activation patterns from one participant for the full week and the mid/bottom of Figure 3A shows the “speed” of one participant’s brain throughout the week: how quickly the brain changed its network pattern between consecutive time windows. Times of high speed occurred in “bursts” where the brain rapidly modulated its networks before stabilizing into a new configuration (examples in Figure 3C; Figure S7 quantifies the “burstiness” of the dynamics across all participants). These results illustrate that brain network dynamics follow a punctuated equilibrium of relatively stable “equilibrium” periods of slowly changing brain networks that are “punctuated” by transitory bursts of rapidly changing brain networks.

To assess how these neural transitions related to behavioral transitions, in nine participants with high-quality video recordings, we marked periods of time when participants underwent three broad categories of behavior: interacting with a digital screen, socializing with another person, or physically manipulating an object. We marked times when participants began or ended one of these three behaviors and found that neural and behavioral transitions tended to coincide with one another (Figure 3B; detailed examination of the relationship between the behavioral and neural states themselves is performed in a later section below).

## Brain networks transitions were circuitous, unpredictable, and chaotic.

How does the brain transition between the starting and ending states of these bursts? Do these bursts take consistent paths? To answer these questions, we defined a neural state-space: a representation of the brain in a high-dimensional Euclidean space where the axes represent the activation of different brain networks. A single time window forms a point in this space where the point’s position along each axis marks the coherence of each brain network during that time window. A transitory burst (a series of consecutive time windows where brain networks start in one configuration and ends in another) becomes a trajectory in this state-space: a series of points leading from a starting state to an ending state.

We found that these bursts were circuitous by measuring the total distance traversed by a trajectory (the trajectory’s length) and comparing it to the trajectory’s displacement (the straight-line distance between the starting and ending point). The distance was on average 8.9 times longer than the displacement across participants during transitions and the ratio for transitions is greater than during stable states (8.9 versus 6.0; Supplemental Figure S13). Examples of these circuitous trajectories from one participant are found in Figure 3C.

After grouping bursts that shared highly similar starting states, we calculated the distance between their trajectories as a function of what percentage of the trajectory was complete. If neural bursts went from their starting to ending points in a consistent manner, bursts sharing the same starting and ending points would take highly similar paths. Instead, nearly the first half of these paths displayed almost as much variability between bursts sharing a destination as between bursts with different destinations despite sharing their starting location (Figure 3D). Only when about 75% of the trajectory was complete did the variability between bursts sharing both a starting and ending point diverge from the variability of bursts sharing only a starting point by a Cohen’s d effect size of one. Using 0–1 chaos tests, we found that chaoticity within the brain’s dynamics rose during times of these transitory bursts (Figure 3E), indicating that these transitory bursts were both non-repeated and chaotic-like. Additionally, the size of these transitions and the time between them followed power laws that are oftentimes associated with chaotic and critical systems (Figure 3F) (20).

During natural behavior, instead of taking a direct, consistent route between neural states, the brain undergoes a chaotic, exploratory-like phase where the variance of its trajectories dramatically rises before stabilizing onto a destination. Upon reaching these destinations, the brain would enter stable states of decreased variability and chaoticity, presumably exploiting currently active networks to accomplish some goal until the participant’s behavior changed once again. These transitions may reflect real-world correlates of task switching typically studied in lab experiments (21, 22) thought to relate to “cognitive flexibility.” These dynamics extend early studies of chaos in the brain and ongoing theoretical models (23–25) based on task data in controlled settings.

## Neural dynamics are driven by a central homeostatic-like attractor at the default mode.

While the brain’s movement appears chaotic, is there a consistent anatomical trend and organization? Do dynamics organize consistently with respect to behavior? System dynamics, in and out of biology, are traditionally defined around their critical points: points in the system that draw in or push out the system’s dynamics (26). If we describe how a ball rolls throughout hills and valleys, we describe how it rolls away from the top of hills and towards the bottom of valleys. In metabolic physiology, we describe sodium or glucose levels by how they fluctuate relative to the homeostatic equilibrium points they stabilize to.

In order to capture the brain’s complex and chaotic dynamics in a simple dynamical form, we used self-supervised deep recurrent neural networks and Koopman operators (27). We started by taking all the data from the week except for two days and learning the underlying building blocks of these dynamics. Neurocognitive states form out of combinations of these individual building blocks and their dynamics unfold according to their interactions and trends, like how words form out of combinations of letters and a sentence’s meaning unfolds from the grammatical interactions of its words and phrases. More specifically, we took the original state-space and mapped each point in it onto a new nonlinear manifold where each axis of that manifold represents a single building block and the brain’s overall neurocognitive state becomes a sum of these blocks. We defined this nonlinear axis transformation such that the temporal evolution of these blocks can be captured using easily interpretable linear methods, allowing us to identify its underlying dynamical drivers.

To validate our model, we took the two days that were not used to learn our neural networks and Koopman operators and annotated the participant’s behavior during these days into the three major categories used in Figure 3B: watching a digital device, socializing, and physically manipulating an object. We trained linear behavioral prediction classifiers on the brain’s position along the learned manifold on one day and tested them on the other day. If learning these building blocks increases classification accuracy, then the building blocks and states learned by the algorithm are neurocognitively relevant.

**Fig. 4.**
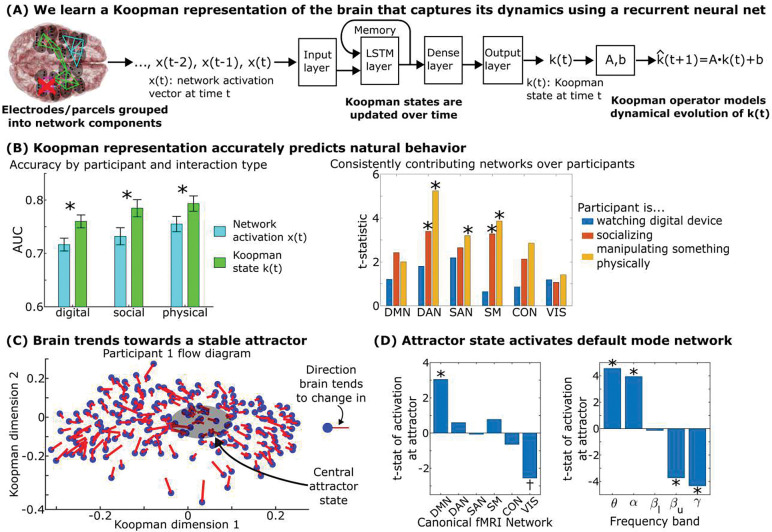
Neural dynamics are driven by a central homeostatic-like attractor at the default mode. (A) We learned a Koopman representation of the brain’s dynamical state by using a recurrent neural network to project the original network activations into a higher-dimensional “Koopman space” where the trajectories of the brain in this space could be captured by linear operators. (B) Trajectories in Koopman space more accurately predicted natural behavior than the original network activations (p=0.012 by paired t-test). Error bars on the left indicate 95% confidence intervals across participants. Anatomical regions consistently activated during each behavior across participants are shown on the right. (C) Flow diagram of how the brain’s state tends to change over time, showing their overall tendency to drift towards a central attractor state which is quantified over all participants in Supplementary Figure S11. (D) At this central attractor state, the brain consistently activates the default mode network at low frequencies across participants (p<0.05 post multiple comparisons correction). The dagger marks a t-statistic that is significant independently (p=0.02) but not significant post multiple comparisons correction. Methods described in Supplementary Section M11–13.

Learning this manifold increased our capability to predict all three natural behaviors (Figure 4B-left, p=0.012). This indicates that a) the manifold contained neurocognitively relevant information that could be decoded by interpretable linear operators and b) natural behavior organized consistently within this manifold. To assess parts of this organization, we asked what brain networks were associated with areas of the manifold tied to each behavior. Social interactions activated dorsal attention and somatomotor networks. Physically manipulating an object activated the dorsal attention, somatomotor, and salience networks (Figure 4B-right). Watching a digital device did not consistently activate any of the brain networks we examined across participants.

We next asked whether there was a consistent dynamical organization for how the brain moved around on this manifold. Using eigendecomposition on the Koopman operator, we found that the brain’s dynamics trended towards a central attractor in every participant. This attractor is visualized in Figure 4C where we plot the flow diagram of the brain’s dynamics: how the brain’s state tends to change as a function of which networks are active/inactive, and their overall tendency is to drift towards a central state. This is quantified by the eigenvalues of the Koopman operator in Supplementary Figure S11, which show that in all twenty participants the brain tended to move towards this attractor. At this attractor state, the brain consistently activated the default mode network while trending towards deactivating the visual network across our participants (Figure 4D). Together, these results demonstrate that neural states organized consistently across participants with respect to both outwardly observable behavior and inwardly observable dynamical trends.

## Neurocognitive states form an hourglass-like shape where the default mode attractor separates waking and sleep.

**Fig. 5.**
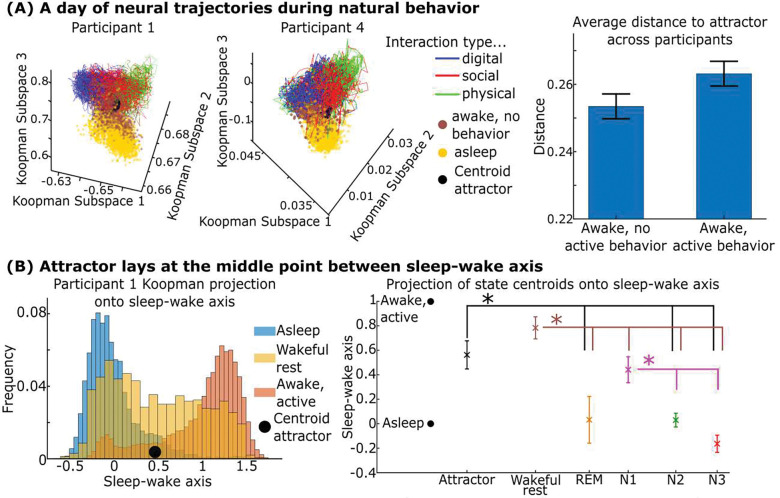
Wakeful rest orbits the central attractor while active behavior departs it. (A-left) This plot shows what brain networks are doing over the course of a day in two participants. Specifically, we plotted a full day of brain network trajectories in the Koopman space, highlighting both the central attractor state along with how different wake states and sleep are positioned relative to this attractor. (A-right) We found that times where the participants were doing one of the three active behaviors tended to depart further away from the central dynamical attractor state relative to times when the patient was awake but not doing any of the three behaviors (p=0.03 by paired t-test). (B-left) We projected neural states from one participant onto the axis separating the centroid of their sleeping and waking states, which we denote the sleep-wake axis. This is equivalent to projecting the hourglasses shown in Figure 5A-left onto a vertical line nearly parallel to the z-axis that runs between the center of asleep and waking states. (B-right) We calculated the centroid of each participant’s neurocognitive states and projected them along their sleep-wake axis. The center of sleeping states was normalized to zero, and the center of actively awake states was normalized to one. We then plotted the centroids’ distribution across participants (error bars indicate 95% confidence intervals). Overhead asterisked bars indicate that the state indicated by the color was significantly different from the marked states by multiple comparisons corrected paired t-tests (p<0.05). We found that wakeful rest and the associated centroid attractor occupied a middle position in the sleep-wake axis with different sleep stages naturally organizing according to their conventionally understood depth. Anatomical regions consistently associated with each sleep stage are illustrated in Figure S12. Methods described in Supplementary Section M14.

If there is a consistent behavioral and dynamical organization of the brain’s nonlinear dynamics, what does this organization look like? How is the representation of different neurocognitive states geometrically organized in this space? In Figure 5A-left, for two participants, we plot a full day of the brain’s trajectory in the Koopman space colored by what behavioral state the participant was in, along with the attractor state. Continuing the analogy from the prior section, this plot is an illustration of how the building blocks of human brain network activity are combined to form the words and sentences of the brain’s language that underpin neurocognitive states participants went through over a day of natural behavior. Qualitatively, these day-long trajectories formed “hourglasses” where different waking behaviors formed separate quadrants in the top of the hourglass, sleep formed the bottom of the hourglass, and periods where the participant is awake but not doing any of the three annotated behaviors formed the middle funnel around the attractor state. We denote this middle funnel associated state as “wakeful rest,” because during these times, participants were awake but not outwardly interacting with their environment.

We verified this structure quantitatively across participants and found that the brain’s state departs further away from the central attractor during times of active behavior than times of wakeful rest (Figure 5A). We defined an axis between the center of brain states associated with sleeping and those associated with being outwardly active and projected the brain’s state onto this “sleep-wake” axis (Figure 5B). Figure 5B-right shows the distribution across participants of the center of various neurocognitive states along this axis, including stages of sleep as determined by an automated sleep score classifier (28). Neurocognitive states were consistently organized with the central attractor and wakeful rest separating actively waking behavior on one end and sleep stages N1, N2, N3, and REM on the other end. The organization of sleep stages in terms of distance from the central attractor matches the conventionally understood “depth” of sleep stages with deeper stages falling further away from the attractor (29). Stages of sleep broadly showed deactivation of anatomical networks across participants: REM sleep was associated with deactivation of the dorsal attention and salience network, while N3 and REM stages displayed several network deactivations that trended towards statistical significance (Figure S12).

Taken together, these results indicate that the default mode network serves an important role as an anchor in the brain’s dynamics. Analogous to how heart rate increases during exercise but will on average have it trend back to resting heart rate via homeostatic forces, the brain’s dynamics trend towards activation of the default mode network. This attractor along with neurocognitive states associated with wakeful rest and N1 sleep formed a central bridge between actively awake behavior and deeper stages of sleep.

Additionally, these findings suggest that long-standing results regarding the default mode network’s role in resting-state fMRI not only generalizes to wakeful rest during natural behavior, but also forms a critical stabilizing anchor in neural dynamics (30).

## Discussion

In this study, we investigated the expression, organization, and temporal dynamics of various neurocognitive states during natural human behavior. By projecting the data into a nonlinear space where locations on this manifold represented the pattern of brain network activations, we found that neurocognitive states formed an hourglass-like structure. Behavioral states clustered in predictable locations along this structure, which allowed us to use network activation patterns to predict specific behaviors, such as whether they were talking to a friend or watching a device. Awake outward behavior formed one end of the hourglass and sleep formed the other end. Times when participants were either awake but not outwardly active or in shallow sleep formed the central funnel.

The brain primarily switched its location along this structure by undergoing sharp bursts of dynamism where it would chaotically explore many areas before settling into a destination. These bursts tended to occur when someone’s behavior was changing, such as when they went from looking at their smartphone to talking to their friends. The dynamics of brain network transitions suggest that when we switch our behavior in the real world, our brains do not undergo a stable, directed shift in neural activity but rather undergo an exploratory-like phase before stabilizing. Despite this chaos, the overall dynamics of the brain tended to be drawn towards a central homeostatic-like attractor located near the central funnel, wherein the brain tended to activate the default mode network and suppress sensory related ones.

Interspersed chaotic-like shifts are seen in other natural and computational systems, such as punctuated equilibrium in evolutionary biology or 1/f avalanches in cellular automata (31, 32). While these systems follow logical rulesets, they periodically generate complex and chaotic system-wide transformations such as phylogenetic explosions. Similarly, bursty transitions between stable states, known as “punctuated equilibrium,” has been proposed as a hallmark of relatively efficient group decision-making (33). The “critical brain hypothesis” argues that emergent complexity in the brain, as in other large, multi-component systems, can occur by amplifying fluctuations on critical boundaries that follow characteristic power law dynamics with bursty characteristics, as we observed here (20, 34, 35). One common theme among these fields is the explore-exploit tradeoff: the concept that many systems incentivized to adapt to changing environments will alternate between exploration-heavy strategies that search for new solutions and exploitation-focused stratagems that fine-tune a single one (36). Our results indicate these frameworks may extend to continuous neural dynamics over longer timescales of hours to days during natural behavior.

Studying brain dynamics at this scale can enable the analysis of cognitive and physiological processes inaccessible on shorter timescales. Our attention, mood, and arousal fluctuate on the order of hours-to-days. Physiological changes such as hormones and gene expression do the same (37). Clinically, many neuropathological states evolve and fluctuate over this timescale. While these fluctuations can be difficult to assess using traditional experimental paradigms, there are ~600 thousand seconds of data in a single week of continuous recordings: 600 thousand examples of the brain’s state in different behaviors, environments, and physiological conditions. Self-supervised deep neural networks, such as the one we used here, offer a rapidly developing method to detect patterns in sparsely labeled data, allowing us to link those patterns more accurately to behavior, physiology, and possibly pathological states. The Koopman operators we used here have seen increasing usage in control theory for their capability to identify underlying drivers of nonlinear system dynamics (38), a critical part of leveraging a system’s natural dynamics during closed-loop control or modulation (9, 10, 39). To help facilitate the use of these methods for other applications, our analysis code can be found at https://github.com/MNobodyWang/WeekLongBrain.

Continuous human brain recordings over a week illustrate that brain networks transition between states via unpredictable and chaotic-like trajectories. These trajectories appear to explore many possible brain states before stabilizing into local states that predictably correspond to behavior, and activation of the “default mode network” during wakeful rest serves as a central attractor for the system. Taken together, these results suggest that the functional flexibility and adaptiveness of our brains are an emergent property (40) of alternations between stable exploitation of specialized local brain states and wide exploration of the brain’s possible configurations during state transitions, showcasing the utility of analyzing continuous neural recordings over long time periods during real-world behavior.

## Supplementary Material

Supplement 1

## Data Availability

Code available on https://github.com/MNobodyWang/WeekLongBrain. Data available on reasonable request.
