# A week in the life of the human brain: stable states punctuated by chaotic transitions

**DOI:** 10.21203/rs.3.rs-2752903/v3

**Published:** 2024-01-15

**Authors:** Maxwell Wang, Max G’Sell, James F. Castellano, R. Mark Richardson, Avniel Ghuman

**Affiliations:** Carnegie Mellon University; Carnegie Mellon University; University of Pittsburgh; University of Pittsburgh; University of Pittsburgh

## Abstract

Many important neurocognitive states, such as performing natural activities and fluctuations of arousal, shift over minutes-to-hours in the real-world. We harnessed 3–12 days of continuous multi-electrode intracranial recordings in twenty humans during natural behavior (socializing, using digital devices, sleeping, etc.) to study real-world neurodynamics. Applying deep learning with dynamical systems approaches revealed that brain networks formed consistent stable states that predicted behavior and physiology. Changes in behavior were associated with bursts of rapid neural fluctuations where brain networks chaotically explored many configurations before settling into new states. These trajectories traversed an hourglass-shaped structure anchored around a set of networks that slowly tracked levels of outward awareness related to wake-sleep stages, and a central attractor corresponding to default mode network activation. These findings indicate ways our brains use rapid, chaotic transitions that coalesce into neurocognitive states slowly fluctuating around a stabilizing central equilibrium to balance flexibility and stability during real-world behavior.

## Introduction

Whether we are fatigued from attending a teleconference call or eager to read a book, whether we feel vibrant and ready to start our day or weary and winding down to sleep, many neurocognitive processes in our lives slowly fluctuate over minutes to hours in chaotic real-world environments. Behaviorally, we transition between tasks like interacting with devices and talking to friends over minutes-to-hours. Neurophysiologically, the interaction between someone’s brain and body is driven by hormones, sympathetic, and parasympathetic drivers related to processes like sleep, arousal, and circadian rhythms that fluctuate over a similar timescale. Yet, most of our understanding of human brain activity comes from well-controlled experiments over short timescales, e.g. studying reactions to carefully chosen stimuli over milliseconds to seconds or examining spontaneous neural activity from subjects “resting” inside a neuroimaging machine. Some studies have longitudinally analyzed brain state dynamics over long timescales by repeatedly sampling a few minutes spread out over days to years using functional neuroimaging^1–11^. A few studies have examined the mesotimescale of minutes-to-hours-to-days in limited settings, such periodically tracking neurophysiological correlates of depression phenotypes^12,13^ or classifying windows of a few circumscribed real-world behaviors^14–16^.

While these studies demonstrate that we can link a short snapshot of the brain’s neural state at single or sparsely sampled points in time to interesting neurocognitive phenomena, little is known about how the brain continuously changes and evolves between these neural states over the mesotimescale of minutes-to-hours-to-days, particularly during natural behaviors that depart from the boundaries of conventional experimental paradigms. Here, we start by identifying the basic dynamical properties of individual areas of the brain and what overall brain states emerge from them. We next ask, how does the brain flexibly change between these states, and how do these changes relate to our behavior and physiology? We finish by asking, are there central stabilizing factors and characteristic brain network modulations that anchor these dynamics?

**Figure 1:**
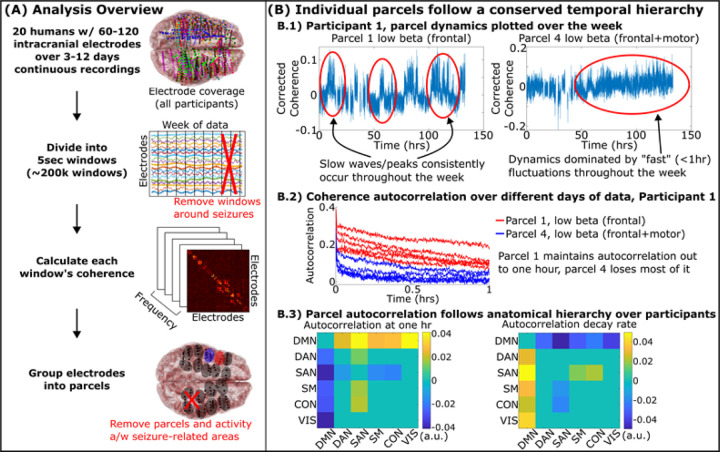
Parcels of the brain followed stable rhythms conserved throughout the week that followed an anatomical hierarchy. A) 3–12 days of continuous recordings from twenty participants were split into five-second-long windows, removing windows around seizure activity and artifact removal. We calculated the coherence between all pairs of electrodes and grouped electrodes with high coherence into anatomically compact parcels. B.1) Coherence within two parcels from a representative participant. B.2) Parcels display unique, stable timescales reflected by their autocorrelation stability over different days of data (all participants shown in Supplementary Figure S3). B.3) Timescale of rhythms between parcels belonging to one fMRI resting network versus another. Cell values indicate the difference in autocorrelation parameter across participants (y axis versus x axis) with positive cells indicating the network indicated by its row has a larger parameter than the one indicated by its column. Non-zero cells indicate statistically significant differences post multiple comparisons by mixed effects model. Methods described in Supplementary Section M6.

To assess human brain dynamics in a real-world setting continuously over days, we leveraged chronic intracranial recordings in neurosurgical participants (80–126 electrodes implanted per participant) undergoing treatment for epilepsy (Figure 1A and Supplemental Figure S1). We examined brain dynamics in twenty humans for between 75 to 283 hours (near-continuous recordings across approximately 3–12 days). During this time, participants were confined to the hospital but would freely socialize with friends, family, and staff, interact with digital devices, sleep, watch TV, and perform other volitional natural behaviors while under simultaneous neural and video monitoring. We found that brain areas and networks would fluctuate and interact in complex and nonlinear fashions that gave rise to neural states that lasted for minutes to hours with chaotic bursts of transitory activity between them. These neural transitions coincided with transitions between behaviors. To identify the underlying driving forces of these dynamics, we turned to self-supervised deep recurrent neural networks and Koopman dynamical system operators. We found that the brain’s mesotimescale dynamics could be split into two categories: a stable set of anatomical networks, relatively preserved over participants, that fluctuated non-chaotically and tracked levels of outward awareness[1] and comparatively rapid, chaotic fluctuations around these networks in a space that tracked changes in behavior.

[1] Here “outward awareness” was operationalized as whether participants were engaged in awake and outwardly oriented behavior, wakeful rest, or, if sleeping, the depth of sleep.

## Results

### Functional parcels showed consistent fluctuations over days and their dynamics displayed consistent anatomic trends.

Before studying how the whole brain evolves over the course of a week, we started by breaking it down into smaller pieces and studying how those pieces change over the week in isolation. We used a data-driven approach to identify small groups of tightly connected electrodes that made up coherent functional brain parcels (we use the term “parcels” rather than “areas” because brain areas are traditionally defined based on anatomical landmarks, and these brain parcels are anatomically compact, but defined based on similarity/high coherence of the neural activity within a parcel). Our first question was whether these parcels showed stable temporal characteristics over the week: e.g., if a brain parcel fluctuates quickly on one day relative to other parcels, does it do so on other days as well?

After removing an hour before and after ictal (seizure) events as determined by the clinical team, we calculated the coherence between all pairs of electrodes in each participant every five seconds over five frequencies: theta (θ: 4–8Hz), alpha (α: 8–12Hz), low beta (β_l_: 14–20Hz), high beta (β_u_: 20–30Hz), and gamma (γ: 30–70Hz)^17^. Electrodes were parcellated into tightly connected, anatomically compact groups of electrodes, each a “parcel” of the brain (Figure 1A) for regional analysis of brain dynamics; later we use coherence between parcels to examine network-level dynamics. After removing parcels and activity associated to the participants’ seizure onset zones, we plotted the activity of each parcel over the week (Figure 1B-top). Fluctuations of these coherences showed characteristic temporal scales that were repeated over different hours and days of data. We quantified this stability by how slowly each parcel’s autocorrelation decayed (timescale). Timescale differences between parcels were stable over time, indicating that parcels that fluctuated faster or slower would remain so throughout the week (Supplementary Figure S3).

Differences between parcels were quantified over all twenty participants by grouping parcels according to which of six canonical fMRI networks they fell in (“default mode”, “dorsal attention”, “salience”, “somatomotor”, “control”, and “visual” as defined in ^18^). Parcels in the default mode consistently showed higher autocorrelation magnitude and longer decay timescales across our participants, whereas parcels of the salience network showed shorter timescales (Figure 1B-bottom). These findings demonstrate a temporal hierarchy separating “fast” and “slow” regions of the brain. A temporal hierarchy, typically measured using autocorrelation, has been hypothesized in the brain with transmodal systems, such as the default mode network, slowly integrating data from faster unimodal regions over seconds to minutes depending on the task^1,2,19,20^. Our results extend these findings to minutes-to-hours in a real-world setting during natural behavior.

### Brain network dynamics predicted physiology.

To assess the neurophysiological relevance of these brain network dynamics, we linked them to fluctuations in circadian rhythms (time of day) and arousal (approximated by heart rate). We used robust principal components analysis to identify parcels and frequencies that covaried with each other, defining each principal component as a “network component” that captured the overall connectome dynamics in a data-driven fashion while reducing the dimensionality of the dataset^21,22^ (we use the term “network components” because the recordings did not have full brain coverage and therefore these covarying parcels are components of brain networks). These network components capture the linear relationships between all parcels, both within frequency bands and across frequency bands, thus assessing both within- and cross-frequency coupling across parcels. The activation of each network component during a window was defined as the weighted average of the parcel coherences within a network component (dot product between network activation and principal component weights). These network component activations showed consistent temporal behavior over time as individual functional regions did (Supplemental Figure S14).

**Figure 2:**
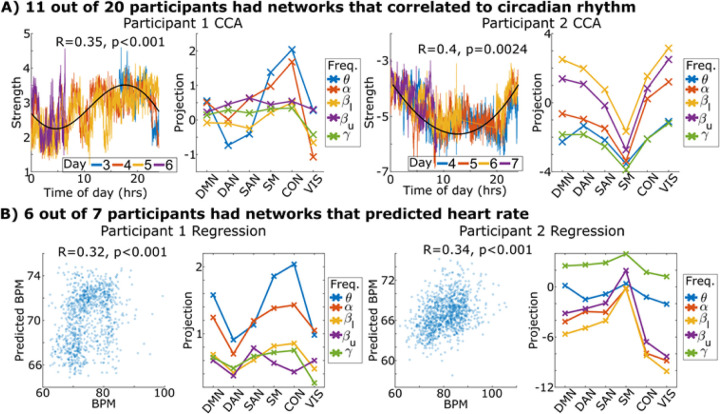
Brain networks predicted physiological markers. A) We linked networks (groups of parcels found using principal components analysis) to circadian rhythm by training canonical correlation analysis on one half of the week and then testing on the other. The network mixture activations during testing are shown on the left plotted against time with the black line indicating a theoretical circadian rhythm. Skips in data are removals due to seizures or disconnected hardware. The identified mixture’s anatomical and frequency coverage are shown projected onto the canonical fMRI networks. B) Networks were linked to heart rate by training linear regressors on one half of the week and testing on the remaining half. Test predictions are plotted against heart rate along with their anatomical and frequency coverage. All participants are shown in Supplementary Figures S5 and S6. Methods described in Supplementary Section M9.

To assess the relationship between brain network dynamics and circadian rhythms, we took the first half of the week for each participant and used canonical correlation analysis^23^ to identify networks that maximized correlation to time of day. We tested this group of networks on the second half of the week using permutation testing and found that 11 of the 20 participants had network components significantly linked with circadian rhythm (Figure 2A; detailed examination of networks consistently contributing to these relationships across participants is shown in Figure 7). Notably, six of the nine participants whose data lacked significant correlation to time of day had sleep disturbances such as nocturnal-awakening seizures or intentional clinical sleep deprivation, suggesting these participants had disrupted circadian rhythms.

Seven participants had sufficiently clean electrocardiogram signals to track heart rate. Heart rate is strongly correlated with the degree of arousal^24^ and is used here as a proxy for it. We used L1-regularized^25^ regression over the first half of the week to identify a group of network components that predicted heart rate and tested this group on the remaining half (Figure 2B). Six of the seven participants had network components that were significantly associated with heart rate.

### Interactions between networks showed consistent, non-linear relationships over time.

**Figure 3:**
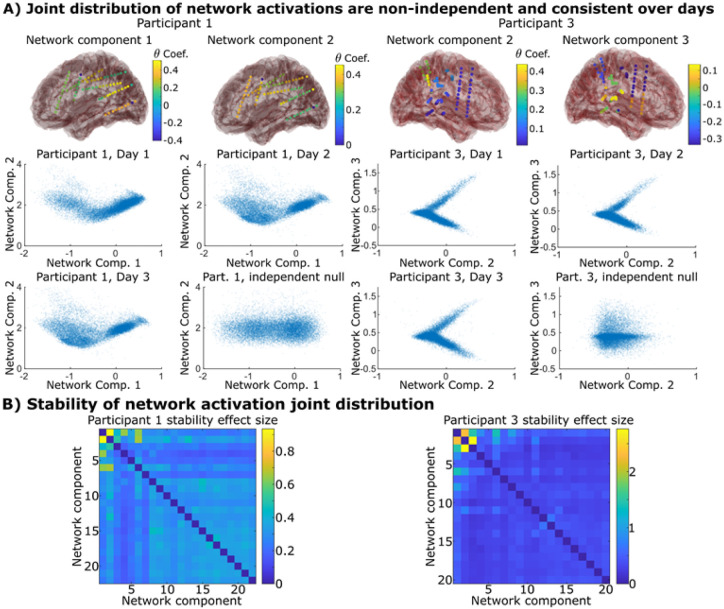
Network components interact in characteristic, non-linear ways that remain stable over the week. A) A pair of network components from two participants that showed non-independent distributions that are conserved over different days of data. The null distribution if the network components were independent of one another are also shown for comparison. B) The distance between the joint distribution of each pair of networks/principal components compared to the null distribution where they are independent of each other. Non-zero effect sizes represent statistically significant distances as determined via permutation testing. We find several pairs that demonstrate such relationships, most notably the lower network components that capture most of the variance in the dataset. Supplementary Figure S13 shows that all twenty participants possessed several such pairs.

Do pairs of networks have reliable interrelationships between their dynamics? We took each possible pair of network components for each participant and asked whether they had a consistent trend in their interactions with one another over different days of data. More specifically, we took each pair of network components and calculated the joint distribution of their activations on each day of data separately. We then assessed whether this joint distribution was both reliably preserved across the week and indicated significant non-independent and/or non-linear relationships (while principal components will group features with linear relationships together, it will not do the same for non-linearities). We calculated the distance between the joint distributions on different days of recordings versus the distance between these distributions and an independent null (more detail in Methods). The joint distribution between brain network components covered characteristic areas in the space that were well preserved over days, indicating that brain networks “dance” with one another in idiosyncratic ways. Some had antagonistic relationships where one network appears to suppress another; some would behave as if one network “gated” the other – inactivity in one network component would mandate inactivity in another. For example, the “V” shaped patterns in Figure 3A indicate that either both networks would be inactive together, or when one was active, the other would either be positively active or negatively active (but not inactive). All participants possessed several network components that showed such pairwise interactions (Figure 3 and Supplemental Figure S13).

### Brain networks underwent bursts of rapid transitions that coincided with natural behavior shifts.

After finding that mixtures of network components were linked to physiology and interacted non-linearly, we examined how the overall network components changed throughout the week. Up to this point, we investigated how parcels, individual network components, and pairs of network components change over time. This is analogous to studying how a hummingbird transitions between hovering and flitting between flowers by only observing their movement in one or two spatial dimensions at a time. Just as how a complete picture of a hummingbird’s flight patterns requires a full three-dimensional space, we studied brain trajectories through a high-dimensional neural space where each dimension was defined by a single network component’s activity.

**Figure 4:**
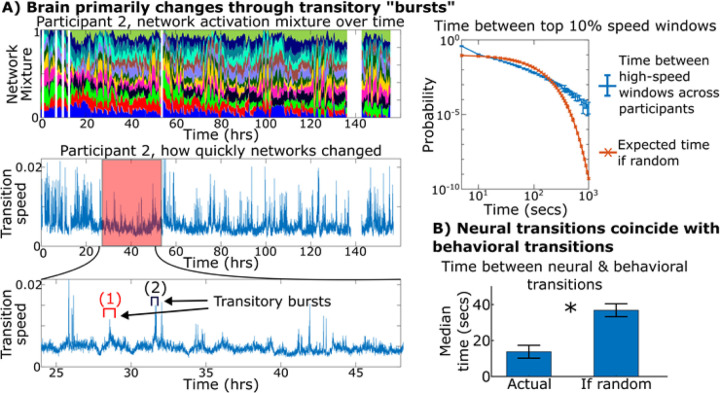
Neural dynamics undergoes bursts of high transitory speed when natural behavior shifts. (A-left) Network activations plotted over the week for one participant where each color represents the activity of a different network (sum of activity normalized to one for visualization purposes) along with how quickly the brain changed network activations every five seconds. (A-right) The average time between windows with the top 10% of transition speed across all participants is shown in blue vs the expected time if windows of high speed occurred via homogenous Poisson process (λ=0.1). Error bars show 95% confidence intervals across participants. We found an increased occurrence of temporally adjacent or near-adjacent time windows of high transition speed, indicating that times of high speed occurred in “bursts” rather than in the distributed manner a Poisson process would indicate. (B) Average time across participants between neural and behavioral transitions compared to the expected time if no relation between the two. Neural and behavioral transitions tended to occur with each other (p=1e-4, paired t-test).

Figure 4A-left shows the brain network activation patterns from one participant for the full week and the “speed” of one participant’s brain throughout the week: how quickly the brain changed its network pattern between consecutive time windows. Times of high speed occurred in “bursts” where the brain rapidly modulated its network activations (brain transitions) before stabilizing into a new configuration (brain states) lasting for minutes-to-hours. Specifically, windows with high speed tended to occur temporally adjacent or close to one another (bursts of high speed) at significantly higher rates than if they occurred randomly via homogeneous Poisson process (Figure 4A-right). This is analogous to the speed of a hummingbird’s movement: bursts of high speed when it’s flying, periods of low speed when it’s hovering. Long periods of stable states interspersed with bursts of high speed transitions is characteristic of “punctuated equilibrium”, an observation that many systems and processes in nature, particularly ones that involve adapting to a dynamic environment, do not undergo steady gradual change but rather periods of stability interrupted by rapid bursts of change^26^.

To assess how these neural transitions related to behavioral transitions, in nine participants with high-quality video recordings, we marked periods of time when participants underwent three broad categories of behavior: interacting with a digital screen, socializing with another person, or physically manipulating an object. We marked times when participants began or ended one of these three behaviors and found that neural and behavioral transitions tended to coincide with one another (Figure 4B; detailed examination of the relationship between the behavioral and neural states, and the brain networks underlying them, is shown in Figure 6).

### Brain networks transitions were circuitous, unpredictable, and chaotic.

**Figure 5:**
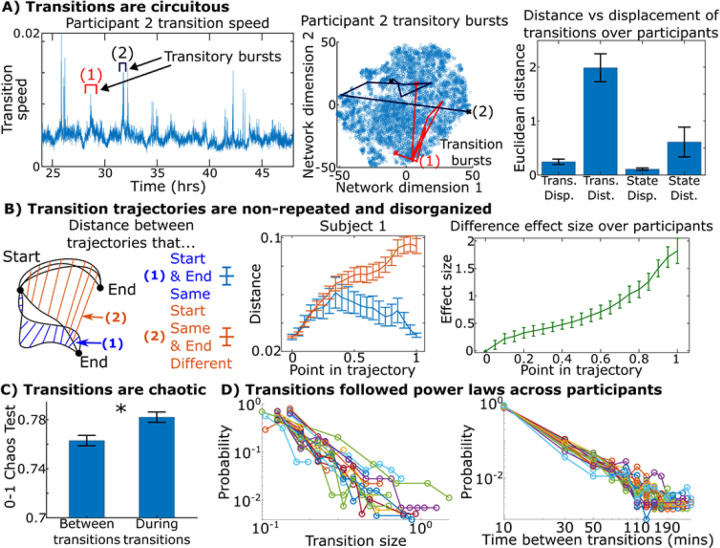
Transitions are circuitous and chaotic. (A-left/mid) Two transitions visualized on a t-distributed stochastic neighbor embedding of the brain’s weeklong course, showing that transitions did not move directly between states but rather explored many interim states. (A-right) Distance (length of the brain’s path during a transition) compared to the displacement (straight-line distance between a transition’s start and end point) across participants. The ratio between distance and displacement during transitions was 8.87±1.19 (95% bound across participants) and 5.99±2.47 for states (p=0.01 that transition ratios were larger, paired t-test). (B) We took transition bursts with the same starting and ending states and asked how similar they were as transitions progressed from start to end (1, blue). We compared this to transitions with same starting but different ending states (2, red). The Cohen’s effect size on the difference between these two distances is shown on the right. The first half of the transitions indicated little about the eventual destination, indicating that transitions in the brain did not take consistent paths from start to end. (C) 0–1 chaos test shows that the chaoticity of brain dynamics rises during transitions across participants (p=1e-3, paired t-test). (D) Distribution of transition size and the time between them for all participants in log-log form. Both distributions formed power laws (linear on log-log axes) across participants statistically tested using Kolmogorov-Smirnov and likelihood tests. Details in Supplementary Section M11.

How does the brain transition between the starting and ending states of these bursts? Do these bursts take consistent paths? To answer these questions, we defined a neural state-space: a representation of the brain in a high-dimensional Euclidean space where the axes represent the activation of different brain networks^27^. A single time window forms a point in this space where the point’s position along each axis marks the activation of each brain network during that time window. A transitory burst (a series of consecutive time windows where brain networks start in one configuration and end in another) becomes a trajectory in this state-space: a series of points leading from a starting state to an ending state. To assess these trajectories, we asked does the brain take relatively straight trajectories when transitioning between states, as a hummingbird does when it moves between one hovering location to another, or are brain trajectories more circuitous? Two such transitory bursts from one participant are shown in Figure 5A-left, where we found that many of these transition bursts did not go directly from one state to another but rather took circuitous routes that explored many interim states before reaching their destination. Visually, these transitions appeared to take very indirect trajectories. Instead of the brain transitioning directly from one stable state to another, it would appear to “wander around” circuitously and explore several possible intermediate states of various network activations or deactivations before stabilizing into the destination.

We quantified this over all transitions across all participants by measuring the total distance traversed by a trajectory (the trajectory’s length) and comparing it to the trajectory’s displacement (the straight-line distance between the starting and ending point). The distance was on average 8.9 times longer than the displacement across participants during transitions and the ratio for transitions is greater than during stable states (8.9 versus 6.0; Figure 5A-right).

While transitions were circuitous, were they consistent? For example, if the brain switched from state “A” to state “B” multiple times during a week, did it take a similar route each time? To assess their consistency, we compared transitions with the same start and end point to transitions with the same start point but different end points (Figure 5B). If transition trajectories were repeated over the course of the week, the distance between repeated pairs of transitions (pairs of transitions that share the same starting and ending states) would remain small compared to the distance between diverging pairs of transitions (pairs of transitions that start in the same state but end up in differing ones). Instead, nearly the first half of these paths displayed almost as much variability between bursts sharing a destination as between bursts with different destinations (Figure 5B). Only when about 75% of the trajectory was complete did the variability between bursts sharing both a starting and ending point diverge from the variability of bursts sharing only a starting point by a Cohen’s d effect size of one (less than one standard deviation apart). Thus, even if two trajectories had the same start and end point, the trajectories were typically very different from each other given that the distance between them was comparable to the distance between trajectories with different ending points for much of their journey.

What are some characteristics of brain network dynamics during these transitions? Using 0–1 chaos tests, we found that chaoticity within the brain’s dynamics rose during times of these transitory bursts (Figure 5C), indicating that these bursts were both non-repeated and chaotic-like. Additionally, the size of these transitions and the time between them followed power laws over minutes-to-hours (Figure 5D). These types of power laws are oftentimes associated with chaotic and critical systems^28,29^. Given that there were often minutes-to-hours between these transitions in the real-world setting, studying these transitions is only possible through neural signals collected not only over extended time periods, but also continuously over such periods.

During natural behavior, instead of taking a direct, consistent route between neural states, the brain undergoes a chaotic, exploratory-like phase where the variance of its trajectories dramatically rises before stabilizing onto a destination. Upon reaching these destinations, the brain would enter stable states of decreased variability and chaoticity, presumably exploiting currently active networks to accomplish some goal until the participant’s behavior changed once again. These dynamics highlight the “explore-exploit” tradeoff in the brain, a key factor for systems that need to adapt to changing environments and goals^30^. Similarly, these transitions may reflect real-world correlates of task switching typically studied in lab experiments^31,32^ thought to relate to “cognitive flexibility.” These dynamics extend early studies of chaos in the brain and ongoing theoretical models^33–35^ based on task data in controlled settings.

### Neural dynamics predict behavior and are driven by a central homeostatic-like attractor involving activation of the default mode network.

While the brain’s mesotimescale state transitions are chaotic and disordered, are there anatomical driving forces behind these neural dyanamics that are consistent across participants? System dynamics, in and out of biology, are traditionally defined around their critical points: points in the system that draw in (stable equilibrium points termed “attractors”) or push out (unstable equilibrium points termed “repulsors”) the system’s dynamics^36^. If we describe how a ball rolls throughout hills and valleys, we describe how it rolls away from the top of hills and towards the bottom of valleys. In metabolic physiology, we describe sodium or glucose levels by how they fluctuate relative to homeostatic equilibrium points they stabilize to.

In order to capture the brain’s non-linear (Figure 3), complex (Figure 4) and chaotic (Figure 5) dynamics in an interpretable dynamical form, we used self-supervised deep recurrent neural networks and Koopman operators^37^. We started by taking all the data from the week except for two days and learning the underlying building blocks of these dynamics. Neurocognitive states form out of combinations of these blocks and their dynamics unfold according to their interactions and trends, like how a sentence’s meaning unfolds from the grammatical interactions of its words and phrases. More specifically, we took the original state-space and mapped each point in it onto a new nonlinear manifold where each axis of that manifold represents a single building block and the brain’s overall neurocognitive state is the combination of these blocks. We defined this nonlinear axis transformation such that the temporal evolution of these blocks can be captured using easily interpretable linear methods, allowing us to identify its underlying dynamical drivers.

To assess whether these dynamics related to participants’ behavior, we took the two days of neural data that were not used to train our deep neural networks and Koopman operators and annotated the participant’s behavior during these days into the three major categories used in Figure 4B: watching a digital device, socializing, and physically manipulating an object. We trained behavioral prediction classifiers on the brain’s position in the Koopman space on one day and tested them on the other day.

**Figure 6:**
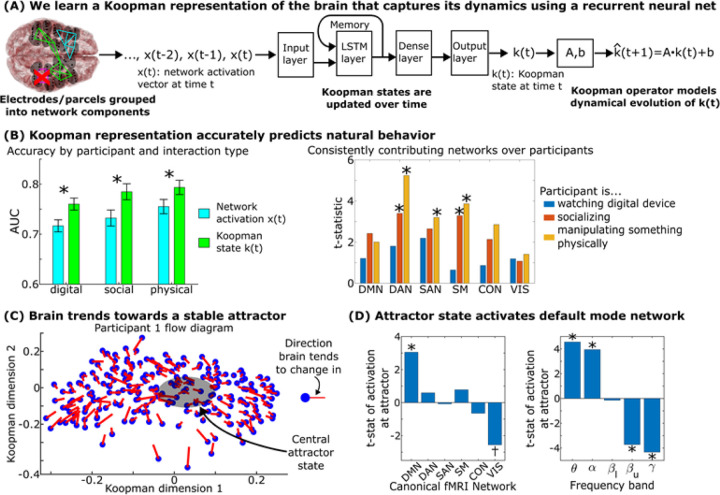
Neural dynamics predict behavior and are driven by a central homeostatic-like attractor involving activation of the default mode network. (A) We learned a Koopman representation of the brain’s dynamical state by using a recurrent neural network to project the original network activations into a higher-dimensional “Koopman space” where the trajectories of the brain in this space could be captured by linear operators. (B) Trajectories in Koopman space predicted natural behavior more accurately than the original network activations (p=0.012 by paired t-test). Error bars on the left indicate 95% confidence intervals across participants. Anatomical regions consistently activated during each behavior across participants are shown on the right. (C) Flow diagram of how the brain’s state tends to change over time, showing their overall tendency to drift towards a central attractor state which is quantified over all participants in Supplementary Figure S11. (D) At this central attractor state, the brain consistently activates the default mode network at low frequencies across participants (p<0.05 post multiple comparisons correction). The dagger marks a t-statistic that is significant independently (p=0.02) but not significant post multiple comparisons correction. Methods described in Supplementary Section M12–14.

The brain state on this manifold predicted all three natural behaviors and furthermore predicted natural behavior better than using the linear mixtures of brain network states prior to applying the Koopman procedure (Figure 6B-left). This indicates that a) the manifold contained neurocognitively relevant information that could be decoded by interpretable linear operators and b) natural behavior organized consistently within this manifold. To assess parts of this organization, across participants, we asked what brain networks were consistently associated with areas of the manifold tied to each behavior. Social interactions consistently activated dorsal attention and somatomotor networks. Physically manipulating an object consistently activated the dorsal attention, somatomotor, and salience networks (Figure 6B-right). Watching a digital device did not consistently activate any of the brain networks we examined across participants.

We next asked whether there was a consistent dynamical organization for how the brain moved around on this manifold (is there a major hill or valley that the brain trends towards?). We found that the brain’s dynamics trended towards a central attractor in every participant. In Figure 6C, we plot the flow diagram of the brain’s dynamics: how the brain’s state tends to change as a function of which networks are active/inactive, and their overall tendency is to drift towards a central attractor state. This is quantified by the eigenvalues of the Koopman operator in Supplementary Figure S11 which show that in all twenty participants, the brain tended to move towards this attractor. At this attractor state, the brain consistently activated the default mode network while trending towards deactivating the visual network across our participants (Figure 6D). There was also an increase in low frequency theta and alpha band activity and a suppression of higher frequency activity at the central attractor state. In physiological systems, central attractor dynamics such as these often reflect homeostatic processes: the tendency for biological systems to equilibrate to central steady points. Here, activation of the default mode network at low frequencies, with suppression of higher frequency activity and deactivation of sensory networks, appears to relate to homeostatic-like processes for the brain. Together, these results demonstrate that neural states were organized consistently across participants with respect to both outwardly observable behavior and inwardly observable dynamical trends.

### Neural states form an hourglass-like shape whose dynamics separate into a physiologically and anatomically preserved center manifold and chaotic fluctuations off of it.

**Figure 7:**
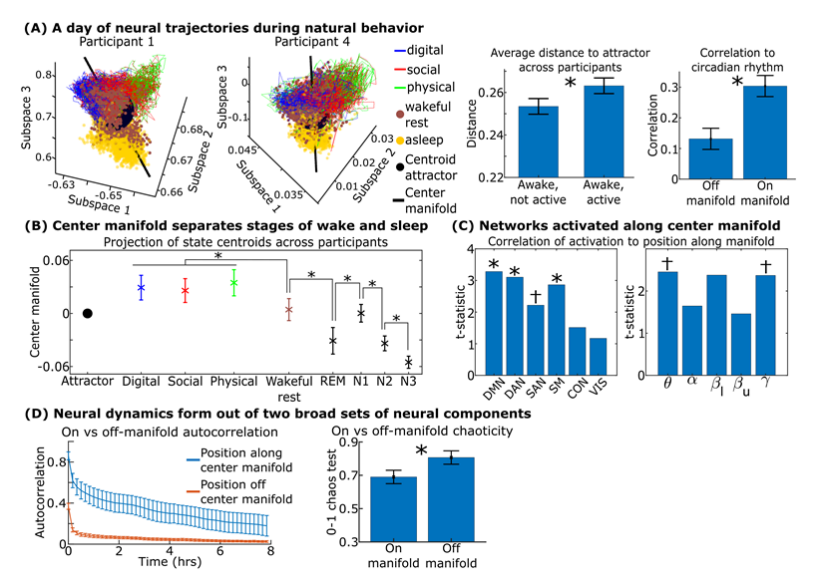
Brain dynamics associated with wake behavior and sleep form an hourglass-like shape consisting of an anatomically stable center manifold and chaotic fluctuations off it. (A-left) A full day of brain network trajectories in Koopman space with both the attractor state and center manifold shown. (A-middle) Times when the participants were doing one of the three active behaviors tended to depart further away from the attractor state relative to times of wakeful rest (p=0.03, paired t-test across participants). (A-right) We separated the brain’s dynamics into two groups: how the brain moves along the center manifold and how it moves around it, finding that its movement along the center manifold was significantly more correlated to circadian rhythm determined via time of day (p<0.01, paired t-test across participants). (B-left) Where the centroid of various behaviors and sleep stages fell along the center manifold with 95% confidence bounds over participants. Asterisks mark neurocognitive states with statistically significantly different centroids along the central manifold across participants (p<0.05, paired t-test, multiple comparisons corrected). For visual simplicity, we only show tests between groups that neighbored each other along the x-axis. Interacting with a device, socializing, and physically manipulating an object did not statistically differentiate along this axis, nor did wakeful rest and N1 sleep, nor did REM and N2/N3 sleep. All other comparisons were statistically significantly different. (C) We calculated the Spearman’s correlation between the activation of the six canonical fMRI networks and five frequency bands and the brain’s corresponding projection along the center manifold. Asterisks mark features with significantly (p<0.05, one-sample t-test across participants corrected for multiple comparisons) different correlations from zero. Daggers mark features that were individually statistically significant but were not significant postmultiple comparisons correction. (D) (Left) The autocorrelation of the brain’s movement along the center manifold and its movement off of it with 95% confidence intervals over participants. (Right) 0–1 chaos tests of these two groups of dynamics. Dynamics along the slow axis dynamics showed statistically significantly less chaoticity than how the brain moved around it (p=0.02, paired t-test). Methods described in Supplementary Section M15.

How do different behaviors and sleep stages organize around this central attractor? Up to this point, we have considered behavior (as measured in broad categories) and physiology (as measured in circadian rhythms and arousal) as two separately analyzed and interpreted quantities, but, in reality, they occur simultaneously. How do their dynamics form together in the context of the chaotic and slow/fast dynamics we have elucidated thus far? Figure 7A-left shows a full day of the brain’s trajectory in Koopman space colored by what behavior two participants were doing, along with the attractor state. Qualitatively, these day-long trajectories formed “hourglasses” where different waking behaviors formed separate quadrants in the top of the hourglass, sleep formed the bottom of the hourglass, and periods where the participant is awake but not doing any of the three annotated behaviors formed the middle funnel around the attractor state. We denote the behavior associated with this middle funnel as “wakeful rest,” because during these times, participants were awake but not outwardly interacting with their environment.

We quantitatively verified the organization of this structure in two ways. We first confirmed that the brain departed further away from the central attractor during times of active behavior compared to times of wakeful rest (Figure 7A-middle). The second was to identify the orientation of behavior and sleep stages along the “center manifold” of the brain. In dynamical systems analysis, the temporal evolution of a system can be decomposed into its constituent parts (eigendecomposition), an important aspect being the “center manifold” which represents the most stable, long-lasting patterns in the system. We plotted the orientation of this center manifold in the two participants in Figure 7A-left and found that it visually aligns as a “sleep-wake” axis. We quantified this by separating the brain’s dynamics into two groups: how the brain moves along the center manifold (up and down the axis) and how it moves off of it (perpendicular to the axis). We calculated the correlation of these dynamics to circadian phase (as previously done in Figure 2A) and found that the brain’s position along the manifold was significantly more correlated to circadian rhythm than its position off of it (Figure 7A-right, p<0.01 by paired t-test across all participants).

We next tested how specific behaviors and sleep stages organized along this manifold in Figure 7B. Across participants, neural states along this center manifold consistently separated into different stages of wake and sleep sorted by their assumed levels of outward awareness and depth of consciousness during sleep: outwardly active waking behavior, times when participants were awake but not outwardly active, shallow stages of sleep, and deeper stages of sleep (as determined by an automated sleep score classifier^38^). All behavior and sleep stages other than shallow sleep (N1) and wakeful rest were significantly differentiated from the central attractor state. Different kinds of active waking behaviors (such as digital vs social interactions) did not separate along this axis even though they separated in the wider Koopman space outside of this axis, as shown in Figure 5B, which includes movement orthogonal to this axis. We found that as the brain moved along this center manifold, it modulated an anatomically broad yet consistent set of networks involving the default mode, dorsal attention, and somatomotor networks across participants (Figure 7C).

This quantitatively verifies across participants that the organization of behavior and sleep stages in the hourglass can be split into two major components. The first component is how the brain moves along this center manifold: how an anatomically broad yet consistent set of networks in the brain fluctuates in ways that tracks levels of outward awareness. The second component is how the brain moves off of this manifold, how brain networks orthogonal to the ones captured by the center manifold fluctuate. Comparing the dynamics of these two primary directions, the brain’s movement off the manifold was comparatively faster and more chaotic compared to its movement along the manifold (Figure 7D).

This is analogous to the movement of clouds. Clouds swirl, interact, and coalesce chaotically, and the particles within clouds transition between rain, snowflakes, hail, and lightning in a chaotic and difficult to predict manner^39^. Despite this chaos, the overall movement of these cloud fronts are guided by the relatively stable prevailing winds and by the Earth’s rotation. This slow orbit (center manifold) slowly and stably tracks broad levels of outward awareness, but it does not separate finer behaviors which emerge as rapid, flexible, chaotic dynamics orthogonal to this axis. In this manner, the brain juxtaposes stable, slow, large-scale dynamics along the central manifold with chaotic, disordered transitions between states to give rise to real-world behavior and cognition.

## Discussion

In this study, we investigated the expression, organization, and temporal dynamics of various neurocognitive states during natural human behavior. By projecting the data into a non-linear space where locations on this manifold represented the pattern of brain network activations, we found that neurocognitive states formed an hourglass-like structure. Behaviors clustered in predictable locations along this structure which allowed us to use network activation patterns to predict specific behaviors, such as whether they were talking to a friend or watching a device. Awake outward behavior formed one end of the hourglass and sleep formed the other end. Times when participants were either awake but not outwardly active, or in shallow sleep, formed the central funnel, characterized by a homeostatic-like stable attractor involving activation of low frequency dynamics and suppression of high frequency dynamics in the default mode network.

The brain primarily switched its location along this structure by undergoing sharp bursts of dynamism where it would chaotically explore many areas before settling into a destination. These bursts tended to occur when someone’s behavior was changing, such as when they went from looking at their smartphone to talking with their friends. The dynamics of brain network transitions suggest that when we switch our behavior in the real world, our brains do not undergo a stable, directed shift in neural activity but rather undergo an exploratory-like phase before stabilizing. Despite this chaos, the overall dynamics of the brain tended to be stabilized by an anatomically broad yet consistent center manifold that tracked stages of wake-sleep related to levels of outward awareness and a homeostatic-like central equilibrium point, wherein the brain tended to activate the default mode network and suppress sensory related ones. This suggests a separation in the brain’s dynamics between 1) how networks broadly track levels of outward awareness in ways that remain consistent across different participants and 2) the ways networks chaotically and unpredictably fluctuate to drive finer changes in behavior which do not remain consistent even within the course of a single week (even though the final stable brain states associated with each behavior does show consistency across days and participants).

Stable brain states interrupted by chaotic-like transitions are akin to punctuated equilibrium in evolutionary biology or 1/f avalanches in cellular automata^40,41^. Evolutionary history does not only show steady and gradual development but also alternates between periods of stability and transient bursts of rapid change^40^. These transitory periods are relatively disorganized: in evolution, these bursts involve phylogenetic “explosions” that generate multiple species or variants that quickly undergo environmental selection^26^. This also generalizes to large human organizations and political systems^42^. In most successful businesses, innovation efforts typically come in waves where an organization will explore several possible opportunities before settling on a much smaller number to develop^43^ which some studies argue is a hallmark of relatively efficient group decision-making^44^. Punctuated equilibrium in brain network fluctuations is also consistent with the idea of metastability in the brain which proposes that dynamic processes with these properties provide the scaffolding for how brain networks engage and disengage with one another^45^. One common theme among these fields is the explore-exploit tradeoff: the concept that many systems incentivized to adapt to changing environments will alternate between exploration-heavy strategies that search for new solutions and exploitation-focused stratagems that finetune a single one^30^.

The power laws associated with this punctuated equilibrium (Figure 5D) are also consistent with the “critical brain hypothesis.” The criticality hypothesis argues that complexity is an emergent property of large, multi-component systems when the system is held on a critical boundary between two self-amplifying behaviors^25^. Maintaining a balance between inhibition and excitation is believed to hold the brain at such a critical boundary^28,46^. Small fluctuations on this boundary are then amplified into larger cascading bursts (or avalanches) of transitory activity which follow characteristic power law dynamics^29,44,47^. For neural processes, this is suggested to yield a balance between maintaining a stable representation of the brain’s current state while remaining flexible enough to rapidly adapt to environmental changes^48^. In artificial intelligence, criticality has been identified in efficient optimization of neural networks^49^.

Studying brain dynamics at this scale can enable the analysis of cognitive and physiological processes inaccessible on shorter timescales. Someone’s attention, mood, and arousal oftentimes fluctuate on the order of hours-to-days. Physiological changes such as hormones and gene expression do the same^50^. Recent technological advances providing the ability to record both neural activity^51^ and physiological biomarkers^52^ in an animal’s home environment can provide a fine-grained view into the cellto-circuit neural behavior underlying cognitive and physiological fluctuations over hours-to-days. In humans, chronic and continuous neural recordings that are performed as standard of care for certain patient populations (including fully natural and deployable recordings in patients with certain deep-brain stimulation systems^53^) can provide the opportunity to study real-world neural behavior on this timescale, both to understand basic neural behavior (as in this study) and to also better understand their pathology. Clinically, many neuropathological states such as dementia, delirium, or acute brain injury responses wax and wane over this meso-timescale^54–56^. Given how many of the brain’s transitions on this timescale occur relatively rarely as rapid bursts of transitory neural activity, perhaps only once every few minutes to hours (Figure 5D), to catch and decipher natural transitions as they occur, neural recordings must be collected and analyzed continuously over extended timescales.

These approaches also allow for an in-depth study of an individual’s neural dynamics. In a single week, there are roughly 600 thousand seconds of data. 600 thousand examples of the brain’s state in different behaviors, environments, and physiological conditions. Self-supervised deep neural networks, such as the one we used here, offer a rapidly developing method to detect patterns in sparsely labeled data, allowing us to link those patterns more accurately to behavior, physiology, and possibly pathological states. The Koopman operators we used here have seen increasing usage in control theory for their capability to identify underlying drivers of nonlinear system dynamics^57^, a critical part of leveraging a system’s natural dynamics during closed-loop control or modulation^12,13,58^. To help facilitate the use of these methods for other applications, our analysis code can be found at https://github.com/MNobodyWang/WeekLongBrain.

Continuous human brain recordings over a week illustrate that brain networks transition between states via unpredictable, rapid, and chaotic trajectories. These trajectories appear to explore many possible brain states before stabilizing into local states that predictably correspond to behavior. Activation of the “default mode network” during wakeful rest serves as a central equilibrium point for the system. This equilibrium point, along with the central manifold that spans levels of outward awareness along a wake-sleep axis, governs slow, stable dynamics of the brain. Taken together, these results suggest that the functional flexibility and adaptiveness of our brains are an emergent property^59^ of alternations over minutes-to-hours during real-world behavior between stable exploitation of specialized local brain states and wide, chaotic, and unpredictable exploration of the brain’s possible configurations during state transitions.

## Data Availability

Code available on https://github.com/MNobodyWang/WeekLongBrain. Data available on reasonable request.
