# Supplementary material for: A week in the life of the human brain: stable states punctuated by chaotic transitions
Source: Res Sq. 2023 Oct 2:rs.3.rs-2752903. Originally published 2023 Mar 30. Preprint. [Version 2] doi: 10.21203/rs.3.rs-2752903/v2 (PMC10081438; doi:10.21203/rs.3.rs-2752903/v2)
Supplement: Supplementary file 1 [file NIHPPrs2752903v2-supplement-1.pdf]

Supplementary Materials for

A week in the life of the human brain: stable states punctuated by chaotic transitions

Maxwell B. Wang, Max G'Sell, James F. Castellano, R. Mark Richardson, and Avniel Singh Ghuman

Corresponding author: mbwang@alumni.cmu.edu; ghumana@upmc.edu

The PDF file includes:

Materials and Methods
Figs. S1 to S12

Materials and Methods

Methods

M1. Participants

Twenty participants (nine males, eleven females; mean age 40 years with a standard deviation of 12 years) had intracranial surface or depth electrodes implanted for the treatment of pharmacologically intractable epilepsy (Figure 1A). All participants gave informed consent to participate under research protocols approved by the University of Pittsburgh Institutional Review Board. Depth electrodes were produced by Ad-Tech Medical and PMT and were 0.86 and 0.8 mm in diameter, respectively. Grid electrodes were produced by PMT and were 4 mm in diameter. Sixteen participants had depth electrodes only, three had grids only, and one had a combination of both. The main results of this paper, when studied on an individual participant by participant basis, did not differ significantly between those with depth electrodes and those with grids.

M2. Analysis overview

In summary, we collected (M3) and preprocessed chronic intracranial recordings continuously over multiple days, which we divided into five second non-overlapping windows, and calculated the functional partial connectome of each window via all-to-all electrode coherence (M4). The electrodes were grouped into tightly connected parcels (M5) and the timescales (autocorrelation) of the parcels determined (M6). We then grouped parcels and frequencies into functional network components using Principal Components Analysis (M7) and studied how the overall mixture of all functional networks would change over time (M9/10). Finally, we learned a dynamical systems model of these networks using a deep recurrent neural network to study the attractor and repulsor dynamics governing brain network dynamics and the relationship between brain networks and behavior (M11-14).

Artifacts were removed at multiple points in the analysis. Specifically, a comb filter was applied to remove line noise (M3). An hour before, during, and after all seizures were removed to eliminate ictal and peri-ictal activity (M4). Spatial regression was used to remove motion, respiratory, and cardiac artifacts (M4). ICA was used to remove large spike artifacts that sometimes occur due to disturbing the cables or connections (M4). Epileptogenic areas and activity that correlated with the activity in these regions was removed to eliminate interictal activity or other pathological activity (M4/5/8).

M3. Intracranial EEG data collection

Electrodes were localized via postoperative MRI or CT scans coregistered to the preoperative MRI using Brainstorm (*41*). Surface/grid electrodes were projected to the nearest point on the preoperative cortical surface automatically parcellated via Freesurfer to correct for brainshift (*42*). Electrode coordinates were then coregistered via surface-based transformations to the fsaverage template using Freesurfer cortical reconstructions. Intracranial electroencephalography (iEEG) data was collected using the Natus system at 1kHz. Notch filters at 60 and 120 Hz were applied with a subsequent bandpass filter from 0.2 to 115Hz.

M4. Data preprocessing and artifact removal

The spatial autocorrelation between an electrode and all electrodes within 2cm of it was then measured and regressed out to remove both local and global artifacts, including artifacts due to motion and current spread due to volume conduction.

Neural activity an hour before and an hour after all seizures, whether electrographic or clinical, was removed before calculating coherence. A board-certified neurologist identified the seizure network in all but two participants, with those two participants having no recorded electrographic or clinical seizures during their stay in the hospital.

The data was then separated into five second non-overlapping windows and coherence was computed over each window between all pairs of electrodes over five frequency bands: theta (4-8Hz), alpha (8-12Hz), low beta (14-20Hz), high beta (20-30Hz), and gamma (30-70Hz). In summary, this generated five connectome structures every five seconds. This was performed using the scipy coherence function under default settings as of version 1.9.3.

Independent component analysis was then applied, and components were visually inspected for any artifacts which were then removed. The main criteria for removal were independent components that possessed time course activations that were clearly non-neurological (such as step-functions or near Dirac deltas).

M5. Parcellation

For each participant, we parcellated their electrodes into groups of tightly coherent electrodes. We utilized the Leiden algorithm to identify a single regional atlas that optimized graph modularity over the entire week-long period across all five frequency bands (*43*). Modularity (Equation 1) was calculated separately over each network from every five-second window with the Leiden algorithm optimizing the average modularity across all windows and frequencies. This generated on average 10-15 parcels for each participant.

$$\text{modularity} = \sum_{b\in\{\theta,\alpha,\beta_l,\beta_u,\gamma\}} \sum_t \sum_{i,j} \left[A_{i,j}^{b,t} - \frac{k_i^{b,t} k_j^{b,t}}{2m^{b,t}} \right] \delta(c_i, c_j)$$

Equation 1: Modularity metric that the Leiden algorithm optimizes. $A_{i,j}^{b,t}$ refers to the weighted connectivity (coherence) between electrodes i and j at time window t and frequency band b. $k_i^{b,t}$ is the degree of electrode i and $m^{b,t}$ is the sum total of all connections at that time and frequency. $\delta(c_i,c_j)$ is an identity function which denotes whether electrodes i and j are in the same parcel. The Leiden algorithm finds the parcellation assignment of each electrode that optimizes modularity over all time windows and frequency bands.

To assess the stability of which electrodes would be grouped into which parcels, we separated the data into six-hour non-overlapping segments (between 18-80 segments per participant) and found the optimal community structure for each segment. We quantified the similarity between each segment's parcel definitions using the Rand Index (*44*) (percentage of electrode pairs that were parcellated equivalently under the two parcel definitions) which almost universally returned values greater than 0.9 as illustrated in Supplementary Figure S2 indicating that the overall parcellation was well-preserved over time. Across participants, this average value had a mean of 0.96 and a standard deviation of 0.02, indicating parcellations remained highly consistent over time. This consistency motivated our decision to use the same parcel structure over the entire work for interpretability.

To ensure that that any potential pathological activity was removed from our analysis, we removed elements of the activity of non-seizure related parcels that appeared to correlate to the activity of parcels within the seizure onset zone. More specifically, using linear regression, we attempted to predict the coherence within non-seizure related parcels using the coherence of seizure-related parcels and then removed this predicted coherence.

M6. Autocorrelation stability (Figure 1)

We tested whether the autocorrelation of each parcel's coherence would show relatively consistent patterns of "fast" or "slow" rhythms throughout the week (Figure 1). We split the entire ~week-long time course for each participant into six-hour non-overlapping segments. After removing parcels associated with the seizure network, we then took the average coherence between electrodes within a single parcel for a single frequency band and then calculated its autocorrelation up to one hour. We fit this autocorrelation curve to a power law ($\mathrm{autocorrelation}(t) = \mathrm{AC}_1 \times \mathrm{time}^{-\mathrm{AC}_2}$) to generate two timescale parameters: AC_1 (autocorrelation strength) and AC_2 (autocorrelation steepness) which described the autocorrelation of a single parcel at a single frequency at a single time segment. For a given frequency band, we took the timescale parameters across all parcels and time segments and grouped the parameters by which parcel they were measured in. We used Kruskal-Wallis ANOVA tests to show that in almost all participants and frequency bands, there were statistically significant differences between the group means, mostly with high effect sizes ($\eta>0.12$, Supplementary Figure S3).

We tested whether parcels from different anatomical regions tended to have reliable differences in their autocorrelation across participants using linear mixed effect models. We assigned each parcel to one of the six canonical fMRI networks defined in (*14*) ("default mode", "dorsal attention", "salience", "somatomotor", "control", and "visual") as based on its largest overlap. For each parcel, we calculated the autocorrelation of its average intra-parcel coherence for a given frequency over the entire week out to one hour and calculated AC1 and AC2 as described above. We then averaged both parameters across all frequency bands.

We then chose a single pair of fMRI networks (such as "salience" vs "visual") and selected all the parcels across our participants that fell into one of those two anatomical groups. We used MATLAB's fitlme (linear mixed effect model) to model each parcel's autocorrelation parameters with the anatomical group as a fixed-effect and the participant as a random effect, allowing us to determine whether one anatomical group had a reliably higher autocorrelation parameter than the other across participants. We repeated this for all possible pairs of fMRI networks and used Bonferroni multiple comparisons correction to identify pairs with significant differences (Figure 1B.3).

M7. Robust principal components analysis

Since many parcels tended to be highly colinear, we used a modified PCA protocol to reduce dimensionality. We grouped parcels and frequencies that tended to covary together using random sample consensus PCA (RANSAC-PCA) on the parcel coherences. By taking the average intra-parcel coherence during each time window and frequency, we formed a (number of parcels x 5 frequency bands) by (number of time windows) 2D matrix which we then reduced to a (number of components) by (number of time windows) matrix using the modified PCA protocol. This identifies parcels and frequencies that tend to strongly covary together that we could easily interpret as a single network component feature that captures cross-frequency

relationships while also reducing the dimensionality of the original dataset to simplify further analyses.

The modified PCA protocol we used applies random sample consensus to avoid PCA's susceptibility to noisy outliers by taking multiple small subsamples of the data and selecting one with the fewest number of outliers to train the model (*45*). We generated 1000 subsamples where in each subsample, we selected six 30-minute segments of data from each day. This ensures that the PCA is robust to rare outliers and that the PCA produces principal components that are stable across days within a participant. Outliers were defined by calculating the Mahalanobis distance between each time window's feature vector and each subsample's distribution. In each participant, we found that these distances would take on clear bifurcations between relatively small distances and short "spikes" of extremely high distances (more than three standard deviations) away from the mean that typically lasted for a few minutes. We manually drew a cutoff for each participant that was approximately half the average Mahalanobis distance of these spikes. For each subsample, we calculated the number of outliers within the subsample, and calculated PCA over the subsample with the fewest outliers. We utilized enough PCs to capture 90% of the variance in the dataset, generally resulting in 12-24 networks/PCs per participant.

The network component activation of a principal component was defined as the projection of the parcel coherences onto the principal component weights.

M8. Seizure network removal

When analyzing parcel dynamics (Figure 1), we excluded all parcels with electrodes part of the seizure onset zone and early propagation as defined by a board-certified neurologist. For network component dynamics (Figure 2 onwards), we first re-added these seizure-related areas before grouping parcels and frequencies into network components through robust PCA. We then removed any network components that were associated with the seizure network before analyzing their dynamics. More specifically, we calculated the dot product similarity between the absolute value of a principal component vector (normalized to a magnitude of one) and a binary vector that marked all electrodes that were part of the seizure network (also normalized to one). The similarity between these two vectors indicated how anatomically similar the driving factors of a principal component and the seizure network were to each other. A null distribution for this similarity was formed by randomly permuting the principal component vectors, and all principal component vectors that showed statistically significant similarity to the seizure network ($p<0.05$) were removed from all further analyses.

M9. Network activation is tied to circadian rhythm and heart rate (Figure 2)

We tested whether we could identify combinations of networks that were associated with neurophysiologically relevant markers. More specifically, we looked at circadian rhythm and heart rate.

Canonical correlation analysis (CCA) was used to identify a mixture of network components that matched a circadian sinusoid with a period of 24 hours. The circadian sinusoid was defined as a1*cos(t/24hrs)+ a2*sin(t/24hrs) where a1 and a2 are constants learned via CCA. CCA simultaneously tried to find a linear combination/weighting of network component activations to fit to this sinusoid.

The model was trained over the first half of the week and then tested on the second half through Pearson correlation (out of sample validation of correlation). The Pearson R of the fit on the test dataset was calculated and then compared to a null distribution of R that was formed via permutation tests that temporally shifted each day's network component activity forward or backwards by a uniform random number ranging from 0-24 hours. This preserves the autocorrelation of the neural signals while eliminating any consistent circadian-like pattern across days.

Heart rate was assessed using collected EKG signals that were processed using heartpy (*46*). The instantaneous heart rate for any window was the average heart rate for a 30-second period centered on the window. L1-regularized regression was trained on the first half of the week to identify a mixture of networks that predicted heart rate using sklearn's implementation (out of sample validation of regression). Hyperparameters were optimized on the training set using ten-fold cross-validation. The quality of the fit was assessed on the remaining half of the week via Pearson correlation with a null distribution formed using the same permutation tests used for circadian rhythm to preserve both the autocorrelational properties of the heart rate and neural signals.

M10. Neural dynamics undergo chaotic-like transitions that are associated with shifts in natural behavior (Figure 3)

We examined how the overall brain state (the status of all recorded networks in the brain) would change over time by dividing the week into "transitions", periods when the brain was rapidly reconfiguring itself; and "states", periods of time where the brain's functional connectome appeared to be relatively stable.

In Figure 3A and Supplementary Figure S7, we provide evidence that the brain falls into states and transitions by examining the "speed" of the brain. Speed was defined as how much the brain's state changed between one five-second window and the next. More specifically, we took the vector of all network activations of each window (the parcel coherences projected into the network PCA space) and calculated the Euclidean distance between the network activation vector of one window and the next (the temporal derivative in the network PCA space).

We calculated the distribution of the time between windows that fell into the top 1% of velocity across all participants and compared that distribution to Poisson distributions with $\lambda=0.01$. The Poisson distribution captures what the expected time between high-speed windows would be in a memoryless process (non-autocorrelated speed). The results showed that windows with high speeds tended to cluster next to each other temporally (supplemental Figure S7). This finding supports the notion that brain network dynamics can be separated into times when the brain is relatively static (states) punctuated by bursts of time when the brain is quickly changing (transitions). Furthermore, we tested this between windows falling within the top 10% of velocity ($\lambda=0.1$) and found the same result. On an individual participant level, we also used Kolomogorov-Smirnov tests to assess whether the time between high transition speed windows followed a Poisson distribution, rejecting the null hypothesis in all 20 participants ($p<0.05$).

In Figure 3D-F, we evaluated various properties of these transitory bursts. We identified neural transition bursts using binary segmentation on the time series of network activation vectors (change point detection), implemented via the ruptures package in Python using default settings (*47*). This algorithm starts by identifying the optimal place to position a single change

point such that the network activations before the change point possess a maximally different distribution from the network activations after it, effectively dividing the week into two pieces. Binary segmentation then recursively subdivides the resulting pieces of time according to the same procedure, until no such change points can be found. Neural transition trajectories were then defined as the time around a change point possessing above average transition speed.

In Figure 3B, we calculated the median time between neural and behavioral changepoints. Using nine participants with available high-quality video recordings throughout the week, we randomly selected two days to annotate for one of three natural behaviors: interfacing with a digital screen, socially interacting with another living creature (either human or in one participant a canine companion), or physically interacting with an object. For this analysis, we excluded times when participants were asleep. In practice, if the participant was not doing one of these three behaviors, they were awake but not outwardly active (wakeful rest/presumably internally thoughtful). Behavioral changepoints were defined as anytime one of these three behaviors started or ended, including times when participants switched from one to another. We calculated the median time between neural and behavioral changepoints and found a null distribution for this metric using permutation testing.

Specifically, we temporally shifted the behavioral changepoints forward or backwards by a uniform random number ranging from 0-24 hours, recalculating the median time difference between behavior and neural changepoints, and used 10000 trials of this to form a null distribution of the expected time difference between behavioral and neural changepoints if there was no temporal relation between the two. We tested whether the real time difference between behavior changes and state changes was consistently smaller than the expected time difference using a paired t-test across participants.

In Figure 3C, we examined the distance between the paths traversed by different transition trajectories. We calculated the distance between the start points of all trajectories in a participant and the distance between their end points. Two trajectories were considered to have similar starting or ending point if the distance between the points fell into the bottom 10% of trajectory pairs. We grouped trajectories into three groups: 1. trajectories with similar starting and ending point, 2. trajectories with similar starting points only, and 3. trajectories with similar ending points only. We calculated the average distance between trajectories that fell into each group as a function of how much of the trajectory had been completed. Specifically, we used linear interpolation to determine what was the brain state 5%, 10%, 15%, 20%, …, 95% of the way into each trajectory. We calculated the distance between brain states of the same percentage in each of the three groups. Figure 3D shows the distribution of these distances for a single participant and the effect size of the difference between these distances across all participants. To determine the effect size, for each participant, we calculated the Cohen’s d between the distances between trajectories that start and end similarly to the distances between trajectories that start similarly but end differently. This measures the number of standard deviations that separate the distributions in the trajectory categories at different points along the trajectory. We then calculated the standard error of these Cohen’s d across all 20 subjects.

In Figure 3E, we studied whether these transitions influenced the chaoticity of the brain dynamics. Chaoticity was defined using the 0-1 chaos test using the protocol described in (*48*) and was calculated over non-overlapping ten minute segments. In summary, we calculated the chaoticity of each network component independently over each time segment. The chaoticity of

the overall neural dynamics for a given time segment was defined as the median chaoticity of all network components. Segments with a transition were compared to segments without one.

$$p(n+1) = p(n) + \phi(n)\cos cn \tag{2.1}$$
$$q(n+1) = q(n) + \phi(n)\sin cn \tag{2.2}$$
$$M(n) = \lim_{n\to\infty} \frac{1}{N}\sum_{j=1}^{N}\left([p(j+n)-p(j)]^2 + [q(j+n)-q(j)]^2\right) \tag{2.3}$$
$$K_c = \lim_{n\to\infty} \frac{\log M(n)}{\log n} \tag{2.4}$$

Equation 2: Chaos 0-1 test protocol. Define *φ(n)* as the network component activation of interest at time window *n* for a given ten-minute segment. This is used to "drive" the dynamical system described in Equations 2.1 and 2.2 where *c* is a randomly chosen "resonance" parameter between 0 and π that remains constant during a single "iteration" of this process. *M(n)* is evaluated up to an *n* of approximately *N*/10 where *N* is the number of time windows in the ten-minute segment. K_c is estimated by fitting a straight line between the numerator and denominator of Equation 2.4 and represents the chaoticity of a single iteration. *c* is redrawn 1000 times and the median K_c is defined as the chaoticity of the network component over the ten-minute segment.

In Figure 3F, we analyzed the distribution of the transition size which we defined as the net displacement of a transition and the time-between transitions. We fit power law exponents to these distributions using MATLAB's nlinfit function with power laws defined as a1*frequency^(-a2) where a1 and a2 are learned.

We tested whether these distributions came from power law distributions using two methods from (*49*). First, we used Kolmogorov-Smirnov (KS) tests to test whether we failed to reject the null hypothesis that the distributions plausibly came from power laws. We fit power law distributions to each participant's transition size and time between transitions distributions separately and calculated the KS distance between the experimental distributions and their theoretical power law distributions. We formed a null distribution on these distances by drawing 10000 random samples from the theoretical power law distribution, fitting a power law distribution to those samples, and then calculating the KS distance between the sampled distribution and the fitted one. If these distances were consistently lower than the distance between the real distribution and its estimated power law one, then we reject the null and conclude that the distribution did not come from a power law. We found that 17/20 participants had transition size distributions that plausibly came from power law distributions ($p>0.05$), and 20/20 participants had time-between transition distributions that plausibly came from power law distributions.

We then used likelihood comparison tests to see whether the transition size and time-between distributions were more likely to have come from power law, exponential, or log-normal distributions. We calculated the log-likelihood that each participant's distributions came from each of the three categories. We used a Wilcoxon signed-rank test to assess whether the log-likelihood of power law distributions were higher than exponential and log-normal distributions across participants. Power law distributions were more likely than exponential

(p=0.007 for transition size and p=4.8e-5 for time-between) and more likely than log-normal (p=0.03 for transition size and p=4.8e-5 for time-between).

M11. Neural dynamics are driven by a central homeostatic-like attractor at the default mode.

To determine whether these brain network dynamics possessed consistent anatomical trends, we used dynamical systems eigendecomposition.

We used a deep recurrent neural network to learn a high-dimensional representation of the network activations where the brain's dynamics could be captured using linear methods. The full model is shown in Figure S9. Our model is an adaptation of the method presented in (*50*) which uses deep neural networks to learn a nonlinear transformation between the original observables of a system onto their corresponding points on a new manifold where the evolution of the system can be captured by linear dynamical laws.

To describe a single forward-pass through the model, we start with x_t, the network activation vector at time *t* (the intraparcel coherences projected onto the robust principal components) for a single participant. This vector was first fed into an encoder, a long short-term memory (LSTM) unit implemented in tensorflow using default settings.

The output of the LSTM was the Koopman representation k_t, a set of variables that summarized the current dynamical state of the brain up to time t. k_t was chosen to have a dimensionality ten times as large as x_t to allow it to serve as a nonlinear kernel. k_t was then passed to two models during training. The first was a linear autoregression model with learnable parameters A, b that attempted to predict the next time step's Koopman state as $\hat{k}_{t+1} = Ak_t + b$. The autoregressive error of this is the squared loss between the predicted and actual Koopman state. In other words, how well does the information encoded in k_t predict its own temporal evolution using linear methods?

We trained all models (*f*, *d*, *A*,*b*) simultaneously according to two loss functions. One of these loss functions asked whether the predicted Koopman state representation at time *t*+1 was close to the actual Koopman state representation at that time. A single training step is described in Figure S10.

We implemented these models using Python's tensorflow Adam optimizer under default settings. All networks used activation functions and L-1 regularization.

M12. Behavioral classification (Figure 4B)

To test whether the Koopman state representation captured neurocognitively interesting information and whether behavior organized consistently along it, we used it to predict the participant's behavior. Participant behavior was manually annotated for three activities: watching a digital screen, socializing with someone else, or physically manipulating an object. These three behaviors were not mutually exclusive. We trained L1-regularized logistic classifiers using Python's sklearn toolbox to predict behavior by training on one day and testing on another (binary classifiers, were they performing the behavior or not). Hyper parameterization was optimized on the training set using ten-fold cross-validation. The area-under-curve of the

receiver-operator-characteristic of each network's ability to classify the desired behavior was calculated.

To determine what brain networks were consistently associated with each behavioral state across participants, for each participant, we selected time windows where the participant was doing a behavior of interest. Using the decoding model, we asked what brain networks were associated with the Koopman state representation of those time windows, forming a (networks x 1) vector for each time window. We averaged these vectors across all time windows associated with the behavior and projected them onto the six canonical fMRI networks. Using the temporal perturbation testing described in our behavioral changepoint testing (temporally shifting the data forwards or backwards by random amounts relative to the behavioral labels), we formed a null distribution for the activation of these six canonical fMRI networks. We then calculated the Cohen's d effect size of the actual average network activation during a task compared to the null distribution with positive effect sizes indicating higher-than-random network activation. Across all participants, we then used one-sample t-tests to see if there was a consistent trend in these effect sizes across participants and used Benjamini-Hochberg for multiple comparisons correction (with the number of tests being 18 for three behavioral tasks over six networks).

M13. Attractor state analysis (Figure 4C)

We calculated the eigendecomposition of the Koopman operator (A from Figure S9). In all subjects, A was full rank and non-ill conditioned, leading to one found critical point (also known as a fixed or equilibrium point): inv(I-A)*b where I is the identity matrix. We found that in all twenty participants, the real component of all eigenvalues had magnitude greater than zero and less than one, indicating this critical point was an attractor (brain states near the attractor tend to be pulled towards it) as shown in Supplementary Figure S11. While dynamical systems may have multiple critical points, this single attractor found by the Koopman operator is conventionally thought to represent the "global" behavior of the system (e.g. a system with both an attractor and repeller will show eigenfunctions associated with the attractor if the system gravitates towards the attractor as time approaches infinity (14)). One caveat is that interpreting systems with more than one critical point using Koopman operators is an active area of investigation (15).

We used the decoding model to ask what original network activation was associated with this attractor state. We projected the resulting network activations onto the six canonical fMRI networks as defined in (16) and averaged over frequency, giving us a six-by-one vector for each participant. We then subtracted each participant's fMRI network activation vector by its mean to ask "which networks were activated or deactivated" relative to the rest of the brain. We used t-tests on each network activation across participants to see if any networks were consistently activated or deactivated. We used Bonferroni for multiple comparisons correction. Participants that did not have electrodes in all six canonical networks were removed from this analysis.

We repeated this process except by averaging over networks to ask if any frequencies were activated or inactivated.

M14. Neurocognitive states form an hourglass-like shape where the default mode attractor separates waking and sleep (Figure 5)

For visualization purposes, in Figure 5A-left, we chose two participants where we could accurately predict all three behaviors and plotted their behavioral trajectories in Koopman space. First, we calculated a subspace where all three behaviors are separated based on the linear classifiers determined in the previous section. The first axis (Koopman subspace 1) was the found feature vector associated with digital screen usage. The second axis was the part of the socialization-associated feature vector that was orthogonal to the first axis. The third axis was the part of the physical manipulation-associated feature vector that was orthogonal to both the first and second axes. We then projected the Koopman state representation from the second (testing) annotated day onto these three axes and plotted trajectories when the participants were partaking in each behavior along with times where the participant was not doing any of the behaviors.

To test whether outwardly active behavior departed from the attractor state relative to wakeful rest (Figure 5A-right), for the nine participants with video annotations, we calculated the distance between each window's Koopman state representation and that participant's attractor state. We then averaged windows based on whether the participant was doing one of the three behaviors or awake but not doing any of the marked behaviors (which in practice meant sitting idly without obvious outward interactions). We used paired t-tests to ask whether the average active behavior to attractor distance was larger than the wakeful rest to attractor distance.

In Figure 5B, we assess the organization of neurocognitive states around this attractor. Specifically, we measure where neurocognitive states tended to form across participants along the axis between sleeping and actively waking states. For each participant, we calculated the mean Koopman state of all windows associated with active waking behavior and did the same separately for sleep. We then defined the vector between these two points as the participant's "sleep-wake axis" and projected the Koopman states from all windows during the annotated days of data onto this axis. Using a sleep score classifier (*28*), we classified windows associated with sleeping behavior into N1, N2, N3, and REM sleep. We then divided windows into the following groups: actively outward waking behavior, waking rest, N1, N2, N3, and REM. We calculated the mean of each group's projection along with the attractor state's projection onto this "sleep-wake" axis. To normalize the range of these projections between participants, we rescaled and recentered these projections linearly such that the center of all sleeping states was at zero and the center of active waking states was at one. We plotted the distribution of these state center projections in Figure 5B, and using paired t-tests with Bonferroni corrections, we assessed whether different states had reliably different locations along the sleep-wake axis across participants. We assessed whether different sleep stages activated different fMRI networks consistently across participants using the same method used to assess for consistent behavioral activation.

Supplementary Figures

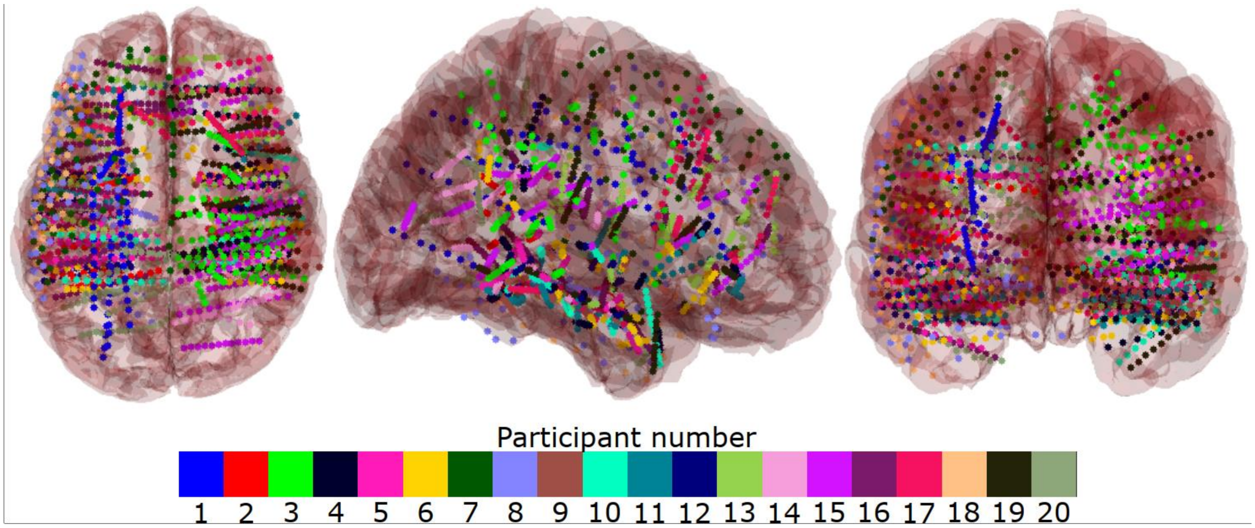

Figure S1: The location of each participant's electrodes in MNI coordinate space. Electrodes with the same color come from the same participant.

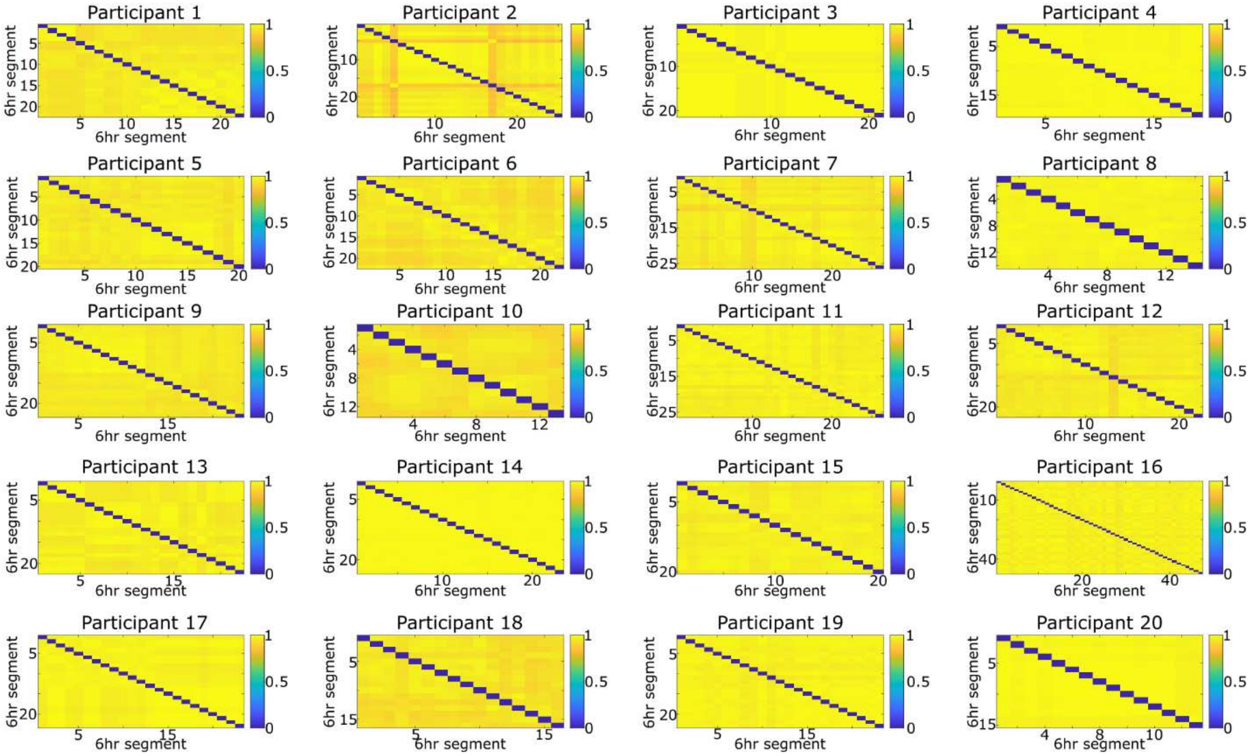

Figure S2: Parcellation stayed steady over time. We divided each participant's data into non-overlapping six-hour blocks and computed the optimal parcellation for each block. The Rand Index, which represents the proportional overlap between two parcellations of electrodes, is shown between all six-hour blocks for each participant. For each participant, we additionally calculated the average Rand index between all pairs of six-hour blocks. Across participants, this average value had a mean of 0.96 and standard deviation of 0.02, indicating parcellations remained highly consistent over time.

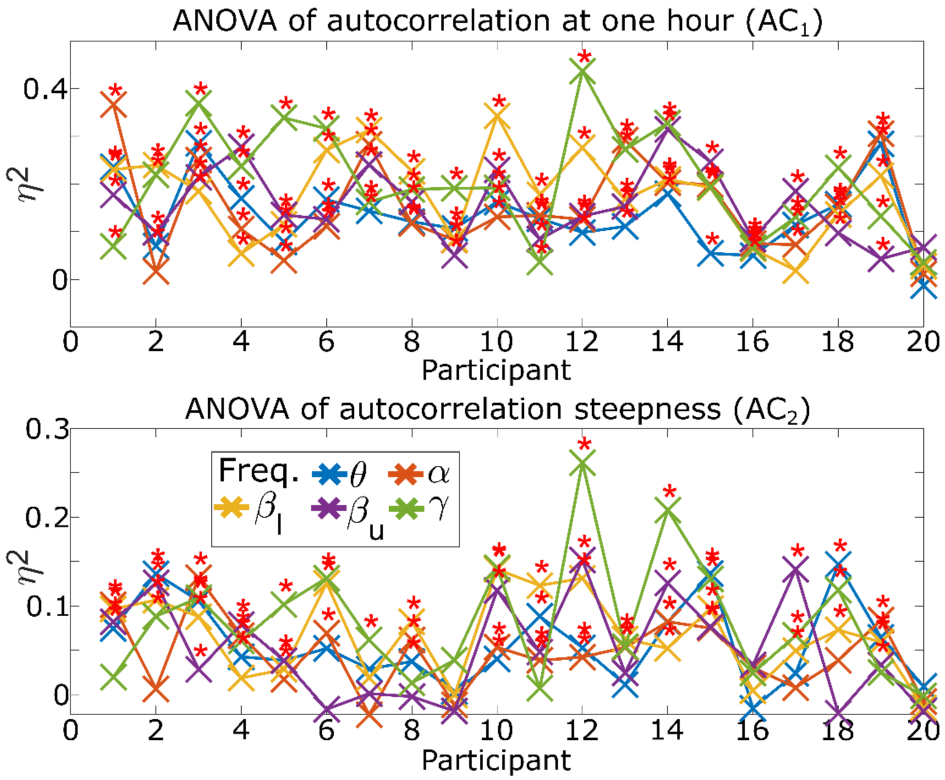

Figure S3: We assessed whether parcels had consistently different timescales using nonparametric ANOVA tests. For each participant individually, we divided their weeklong time-course into six-hour non-overlapping blocks. We calculated the autocorrelation of each parcel's coherence at a given frequency band (θ: theta, α: alpha, βl: low beta, βu: high beta, γ: gamma) across all blocks. We then tested whether the parcels from a single participant and frequency band had different autocorrelations from each other over these blocks using a Kruskal-Wallis one-way ANOVA test. Each group in the ANOVA test was the autocorrelations of a single parcel across all blocks, and we tested for whether there were differences in the group means. The effect size of the ANOVA test is shown above with asterisks marking statistically significant differences ($p<0.05$). η2 effect size indicates the percentage of variance in autocorrelation that is explained by which region autocorrelation was measured in.

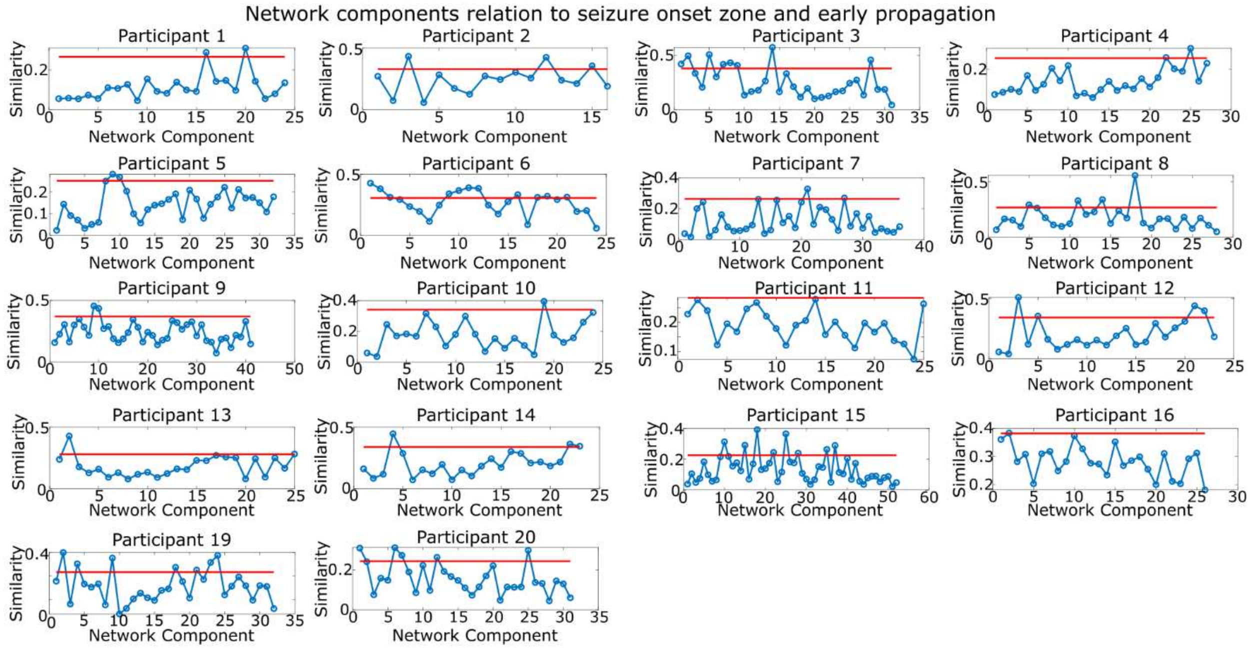

Figure S4: To ensure we remove all network components related to the participant’s seizure related areas, we calculated the similarity between each participant’s network component and their seizure zones and removed any network components showing above-chance similarity. Seizure zones were defined as any electrodes marked as part of the seizure onset zone or early propagation. Similarity was defined as the dot product between the absolute value of each participant's network component and their seizure zone and is shown above for all 20 participants. Participants 17 and 18 did not have any clinically defined seizure network. A null distribution for the dot product similarity generated by randomly permuting each network is shown with the red line to denote statistical significance threshold (p=0.05). All network components with significant similarity to seizure related regions were removed for all analyses on network components (Figure 2 and onwards).

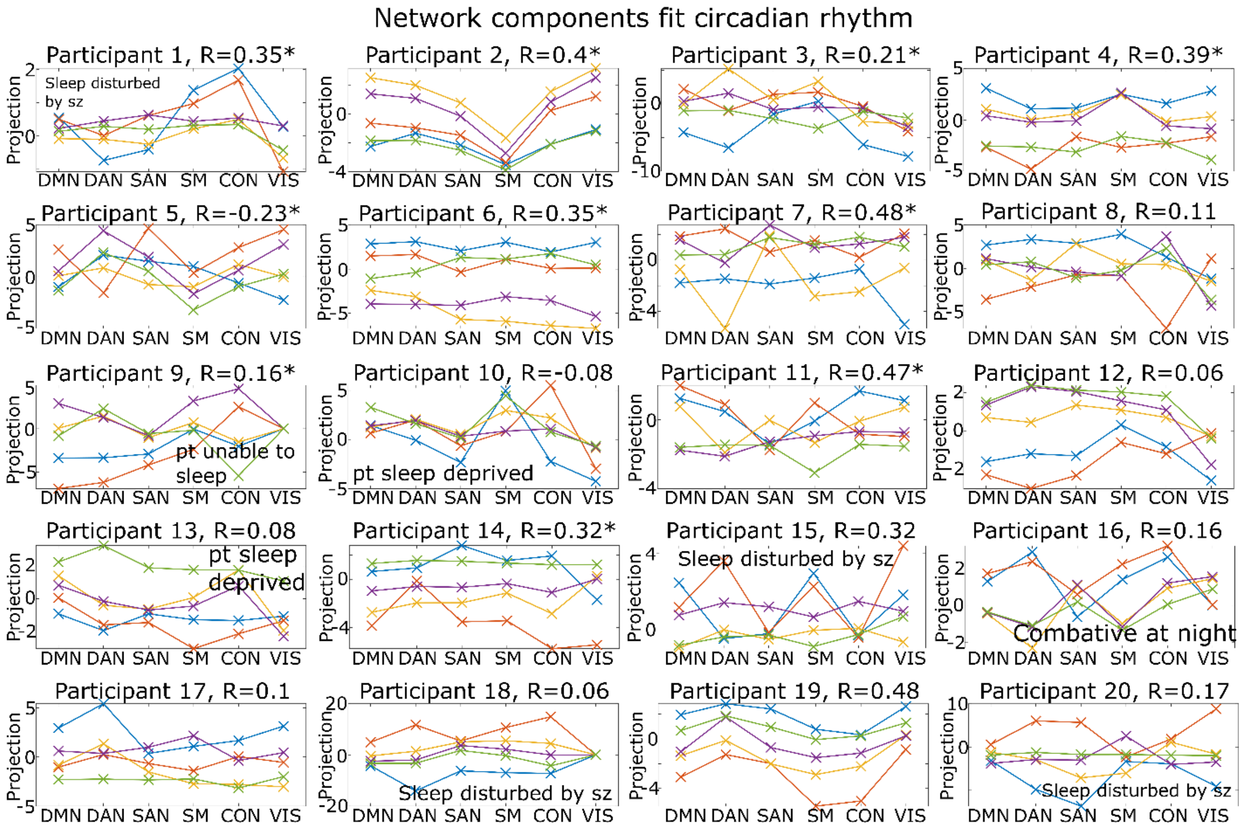

Figure S5: This figure shows the results in Figure 2A for all participants. The anatomical and frequency coverage of the mixture of networks associated with circadian rhythm in each participant are shown above. The correlation between a theoretical circadian sinusoid and the mixture's activation is shown above each plot (R). Asterisks indicate statistically significant correlations.

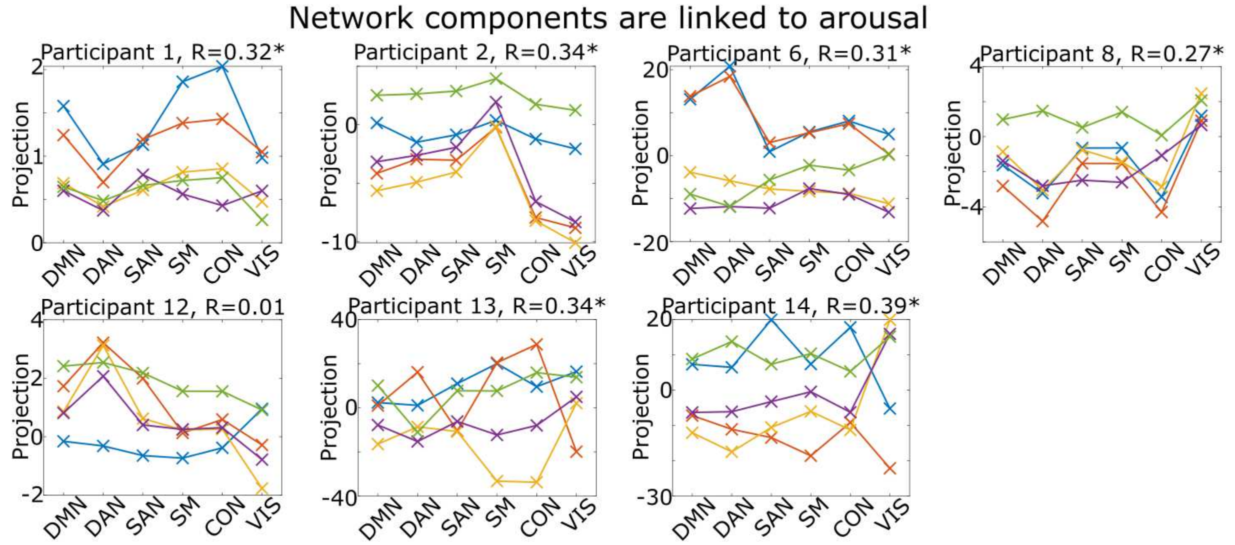

Figure S6: This figure shows the results in Figure 2B for all participants with recorded EKG. The anatomical and frequency coverage of the mixture of networks associated with heart rate (which is used as a proxy for arousal) in each participant are shown above. The correlation between the

heart rate and the mixture's activation is shown above each plot (R). Asterisks indicate statistically significant correlations.

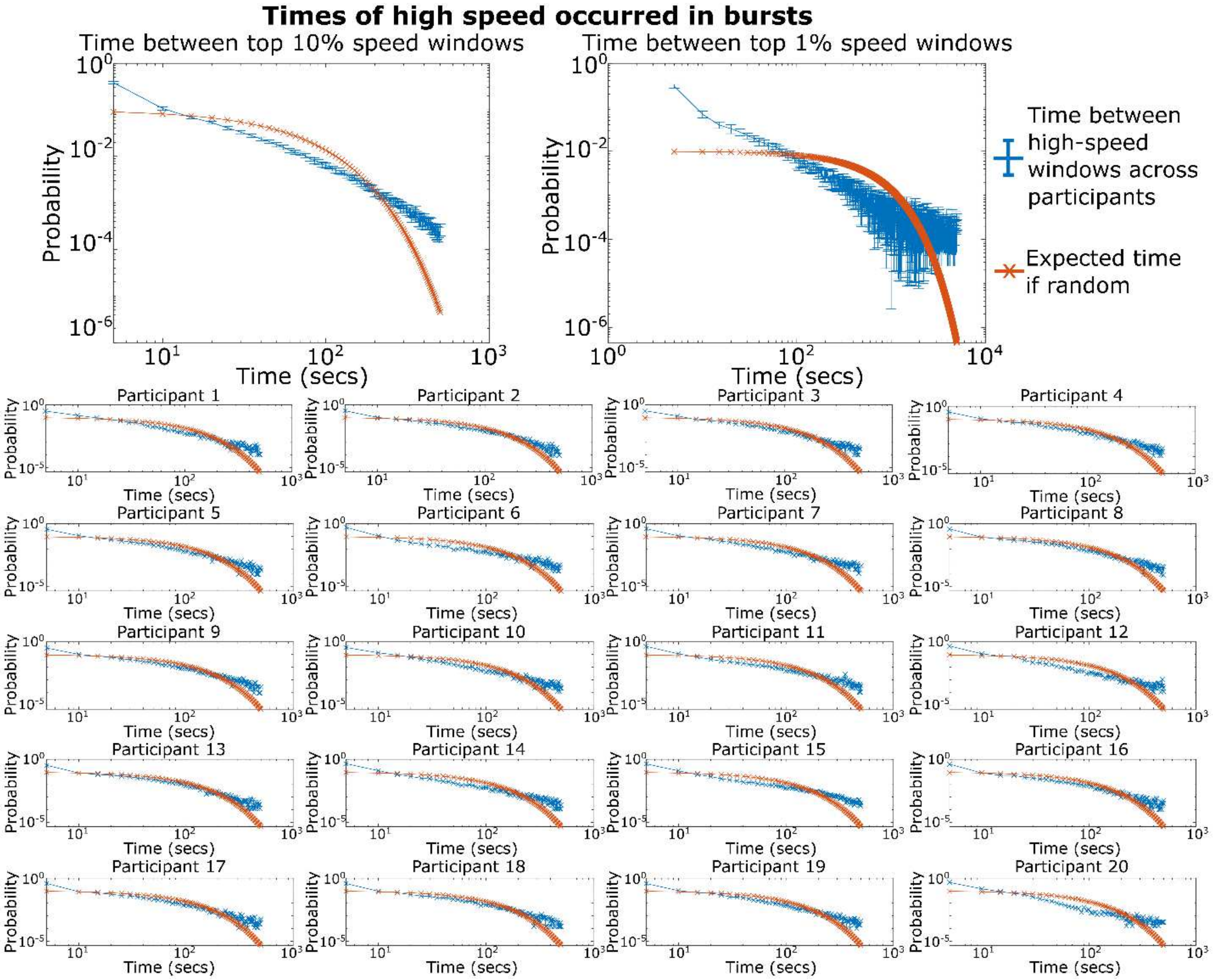

Figure S7. Top) The average time between windows with the top 1% or 10% of transition speed across all participants is shown in blue vs the expected time if windows of high speed occurred via homogenous Poisson process (λ =0.01,0.1). Error bars show 95% confidence intervals across participants. We found an increased occurrence of temporally adjacent or near-adjacent time windows of high transition speed, indicating that times of high speed occurred in "bursts" rather than in the distributed manner a Poisson process would indicate. We also found an increased occurrence in periods of time lasting for several minutes or longer with no times of high speed compared to what would be expected with a random Poisson process, indicating the existence of "stable states" of decreased speed. Bottom) The average time between windows of high speed for each individual participant. The distribution of each participant's time between windows did not follow a Poisson distribution by Kolmogorov-Smirnov test ($p<0.05$ in all cases).

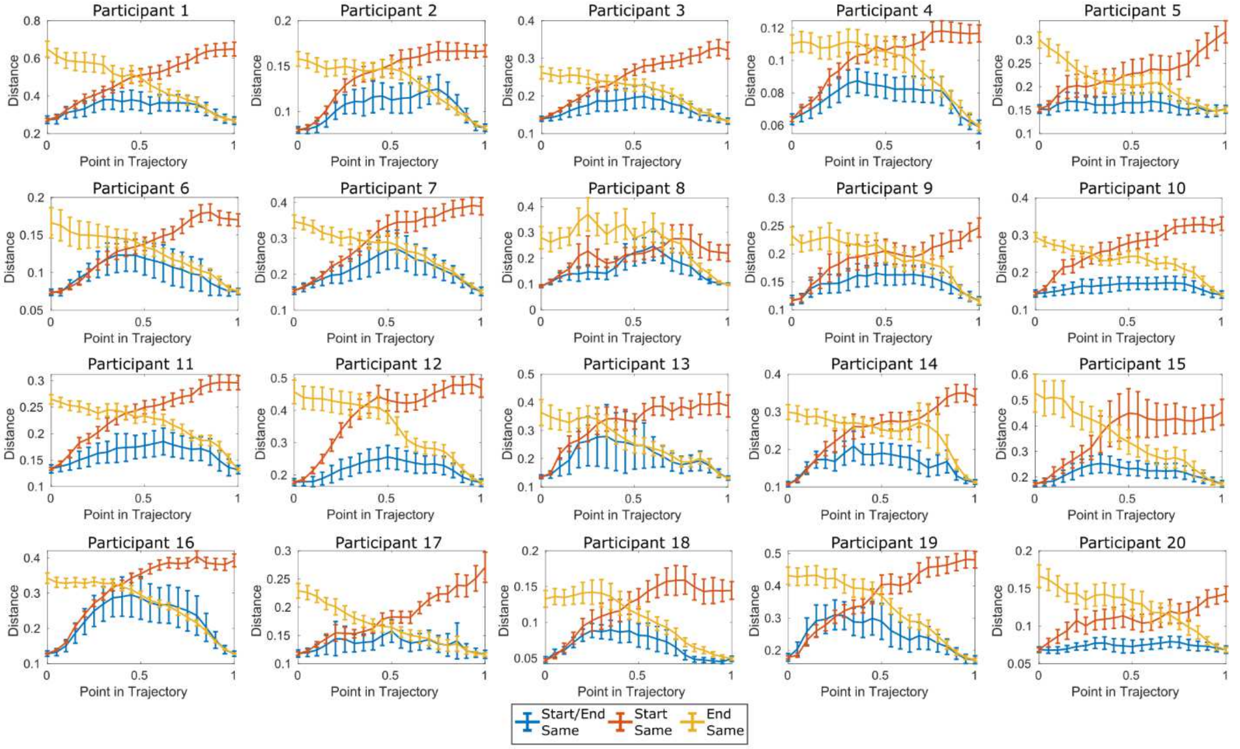

Figure S8: This figure shows the results in Figure 3D for all twenty participants. Average distance between pairs of transition trajectories as a function of what proportion of the trajectory was complete. Trajectory pairs are grouped into three categories: transitions with similar starting and ending points (1, blue) vs similar starting but different ending points (2, red) vs different starting but similar ending points (3, yellow).

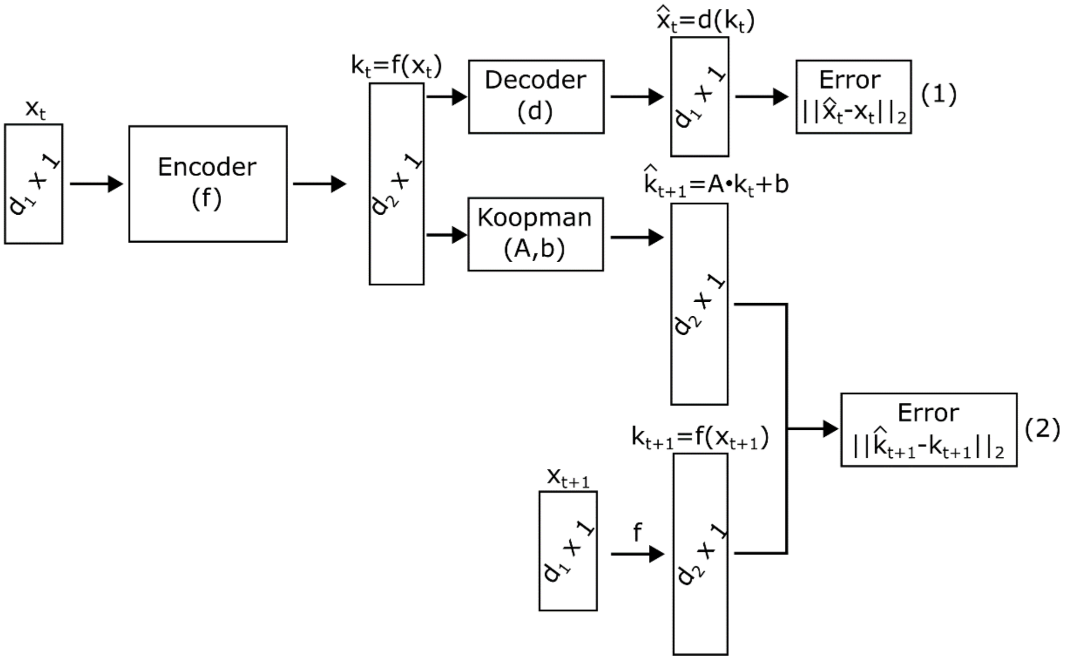

Figure S9: The Koopman model described in Figure 4A. x_t represents the network activations at time t. k_t is the output of the encoding model as is the Koopman state representation at time t.

Here the encoding model is a recurrent neural network, the decoding model is a standard neural network, and the Koopman operator (A,b) is a first-order discrete differential equation.

Algorithm 1 Training step

Feed the first half hour of $x_1, x_2, \ldots, x_{30\text{min}}$ into the LSTM encoder f to initialize its internal state
Define $t = 30\text{min} + 1$ where x_t is the time window right after the initializing half hour
Define $k_{t-1} = f(x_{t-1})$, the output of the LSTM on the previous network activation state
Define the current error to be $\epsilon = 0$
while t <total number of time windows **do**
    $k_t \leftarrow f(x_t)$
    $\hat{x}_t \leftarrow d(k_t)$
    $\epsilon \leftarrow \epsilon + ||\hat{x}_t - x_t||_2^2$
    $\hat{k}_t \leftarrow A \cdot k_{t-1} + b$
    $\epsilon \leftarrow \epsilon + ||\hat{k}_t - k_t||_2^2$
    $t \leftarrow t + 1$
end while
$\epsilon \leftarrow \epsilon / t +$ L-1 regularization term of all learnable parameters
Update f, d, A, b according to the gradient of ϵ w.r.t learnable parameters

Figure S10: A single training step of the overall algorithm described in Figure S9.

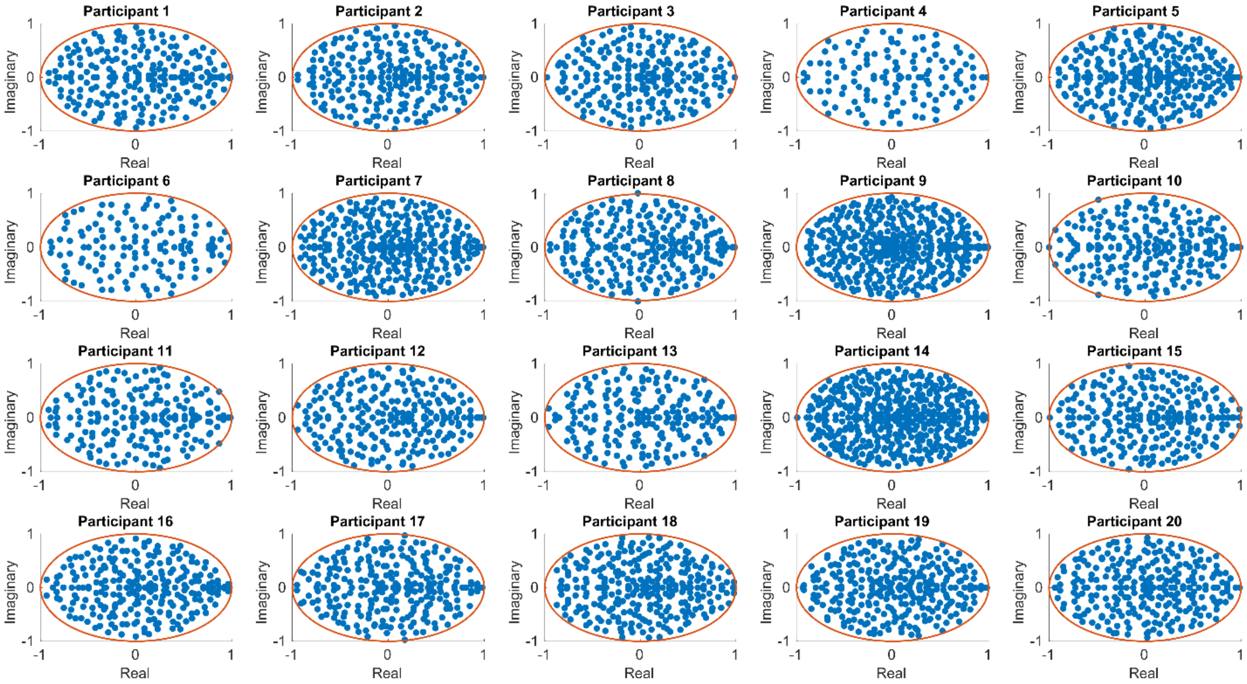

Figure S11: Eigenvalues of the Koopman operator (eigenvalues of the matrix A in Figure S10) over all twenty participants. In all participants, the real part of all eigenvalues is less than one, indicating that the overall dynamics were governed by stable attractors.

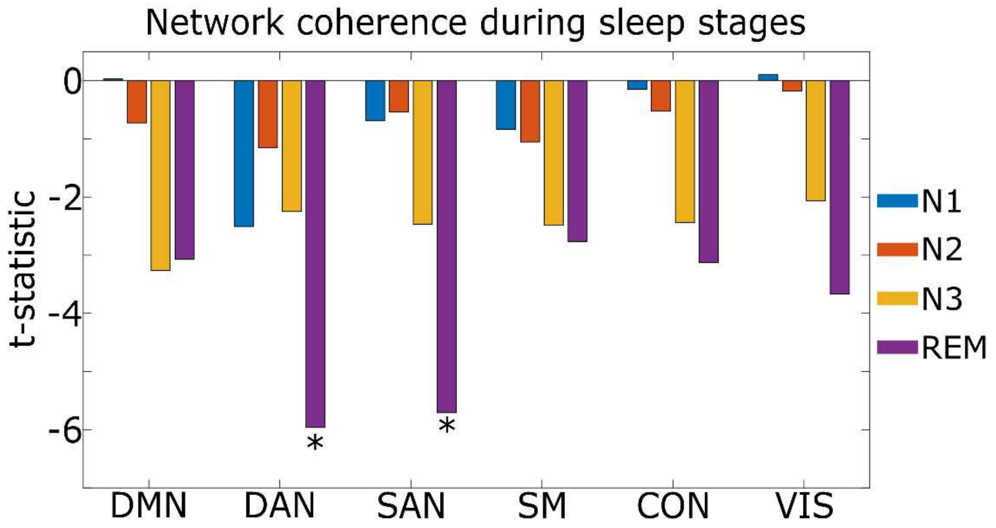

Figure S12: Anatomical regions consistently activated during different stages of sleep across participants. Negative t-statistics indicate that brain networks spanning the indicated fMRI network regions have lower coherence during the relevant sleep stage compared to other time windows throughout the week. Asterisks mark statistically significant networks post multiple comparisons.

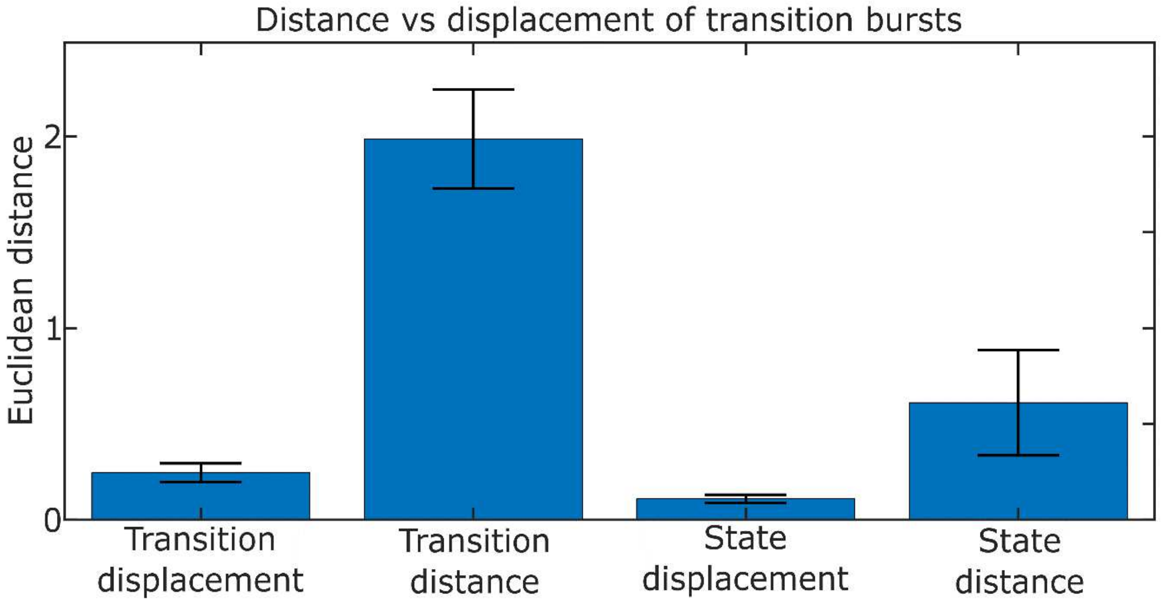

Figure S13: We tested whether brain transitory bursts went directly from one state to another or whether they took indirect, circuitous routes. We plotted the average total distance (sum of transition speed) traveled during transitions and stable states for all twenty participants versus their net displacement (distance between start and end states). Net distances during transitions were 8.87±1.19 times larger than the net displacement (confidence interval indicates 95% bounds on the average multiplier across all participants). Net distance versus displacement during states were 5.99±2.47 with the ratio between distance and displacement were higher for transitions than states (p=0.01, paired t-test).
